# Supplementary figures and images for: SETD2 suppresses tumorigenesis in a KRASG12C-driven lung cancer model, and its catalytic activity is regulated by histone acetylation
Source: eLife. 2025 Sep 15;14:RP107451. doi: 10.7554/eLife.107451 (PMC12435893; doi:10.7554/eLife.107451)

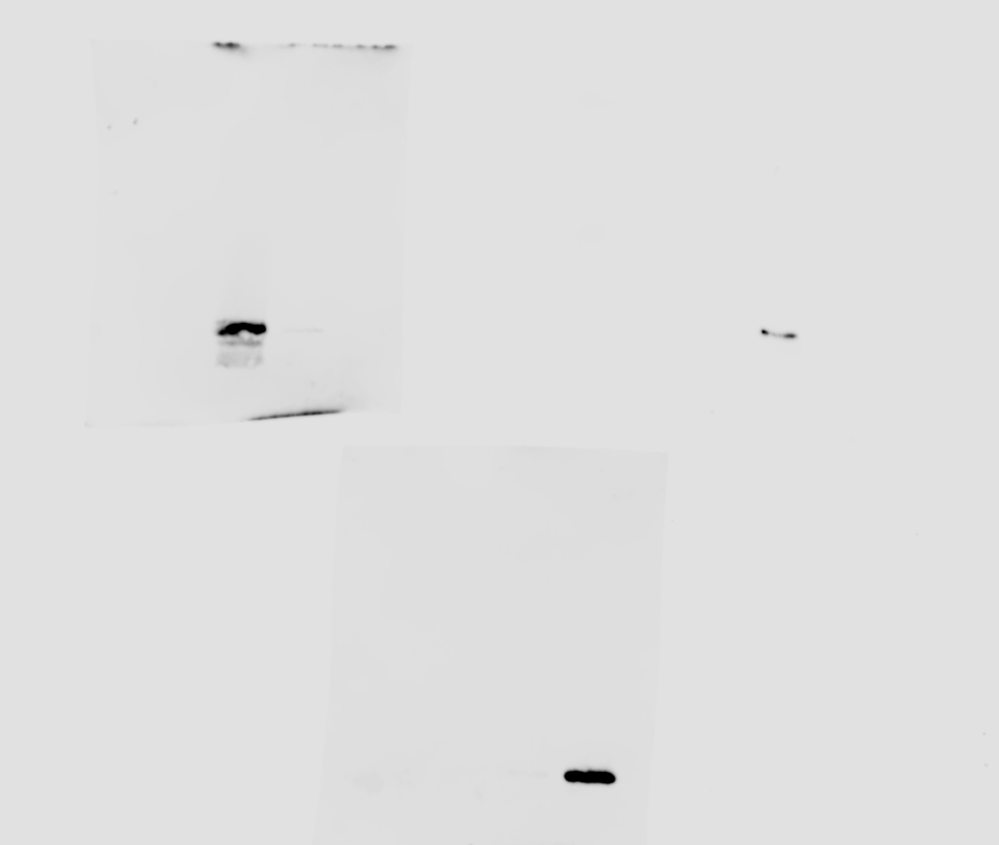

Supplement: Figure 2—source data 1. [file elife-107451-fig2-data1.zip › Figure 2_Source Data/Fig2Eme1.png]

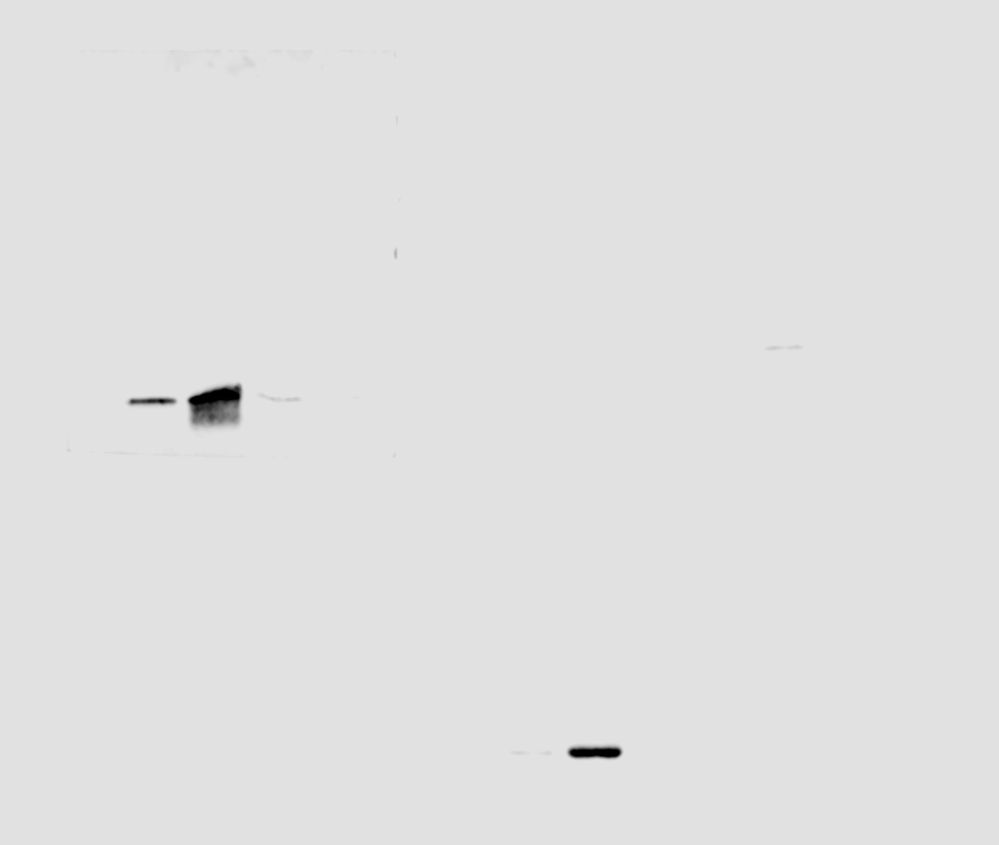

Supplement: Figure 2—source data 1. [file elife-107451-fig2-data1.zip › Figure 2_Source Data/Fig2Fme1.png.png]

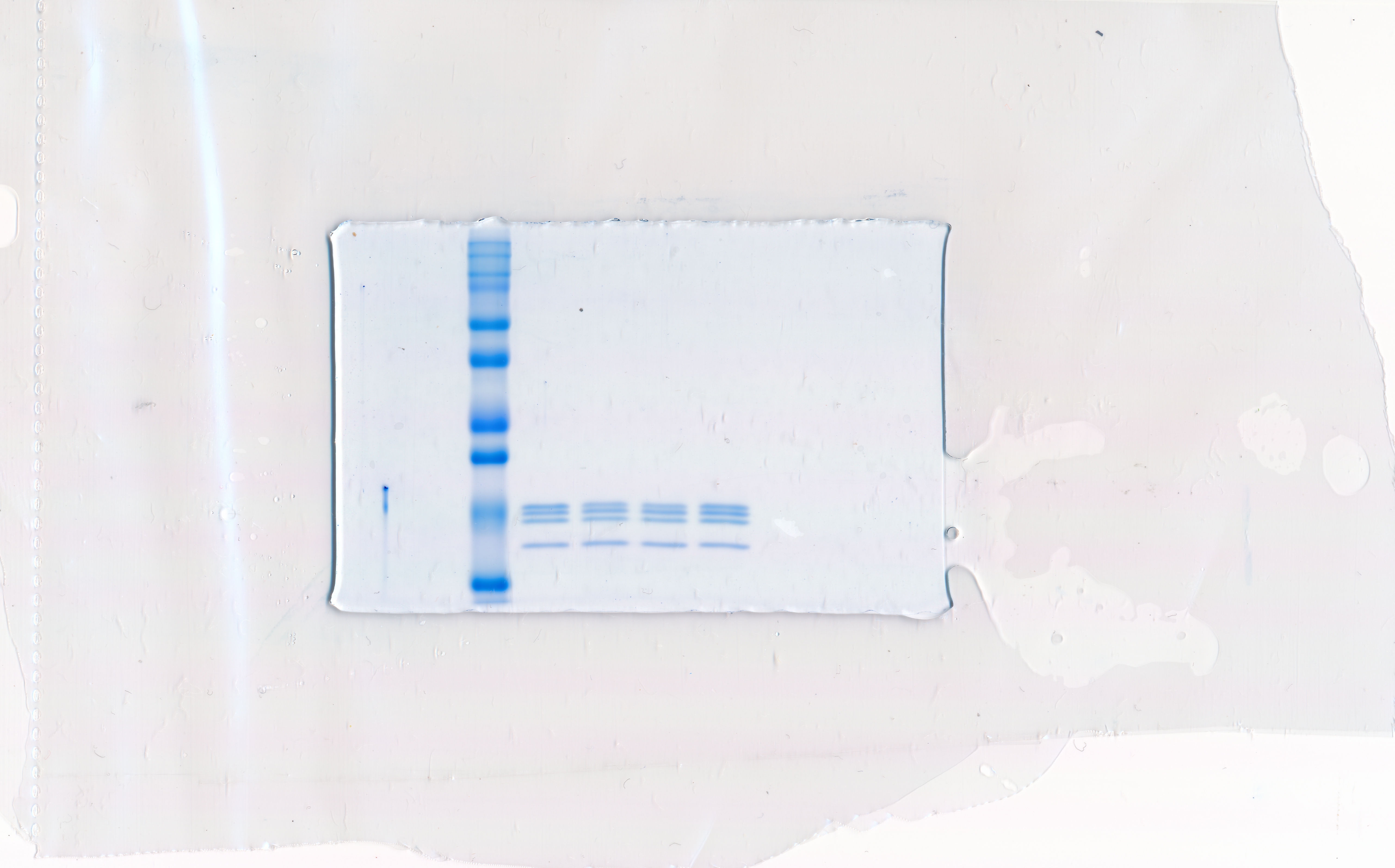

Supplement: Figure 2—source data 1. [file elife-107451-fig2-data1.zip › Figure 2_Source Data/Fig2Bk36me_C_121624_20241217_0001.tif]

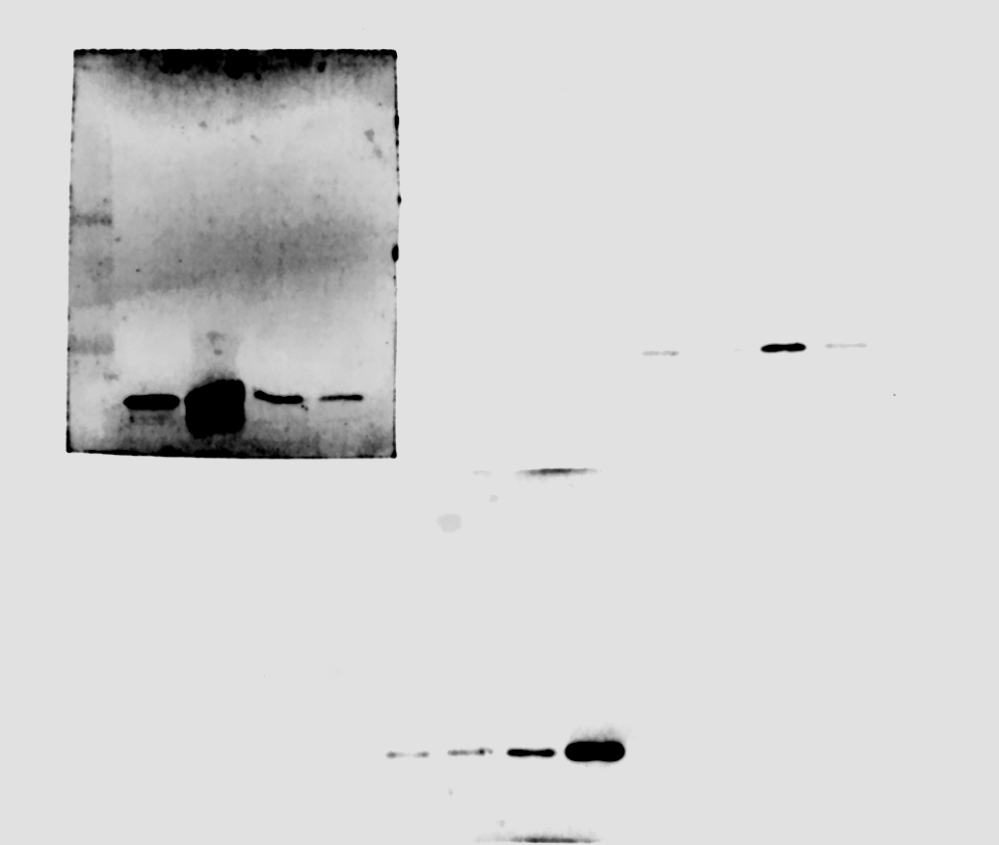

Supplement: Figure 2—source data 1. [file elife-107451-fig2-data1.zip › Figure 2_Source Data/Fig2Fme2me3.png.png]

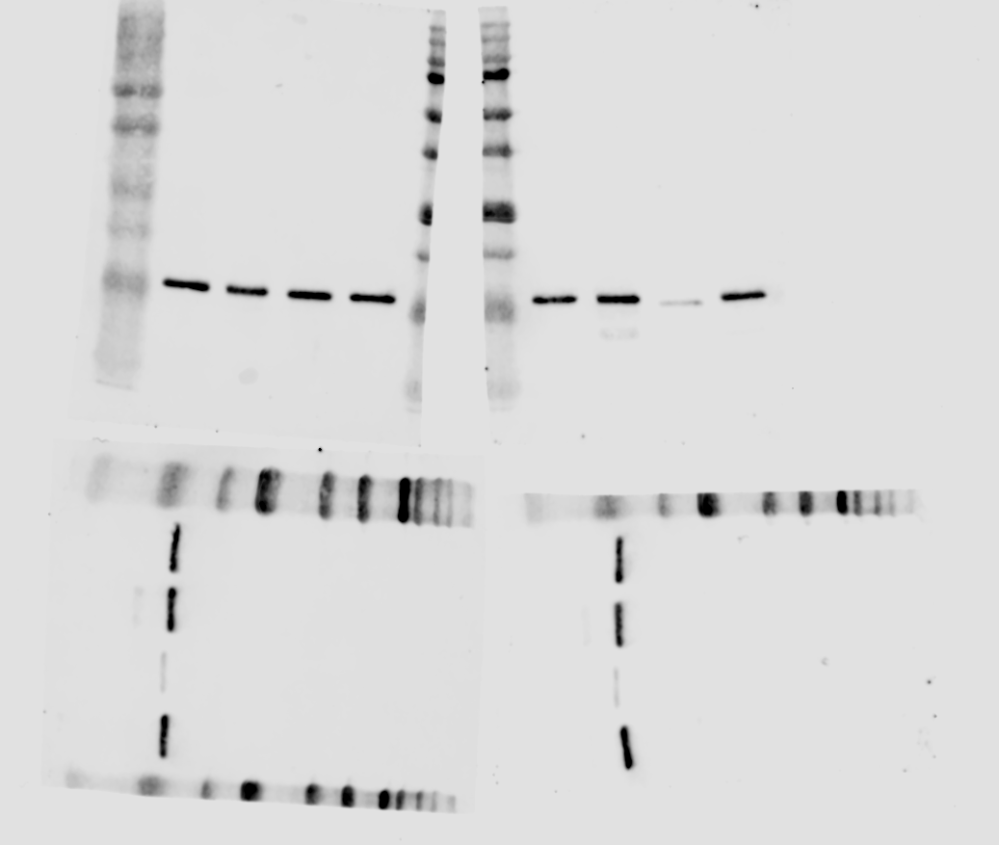

Supplement: Figure 2—source data 1. [file elife-107451-fig2-data1.zip › Figure 2_Source Data/Fig2EH3.png]

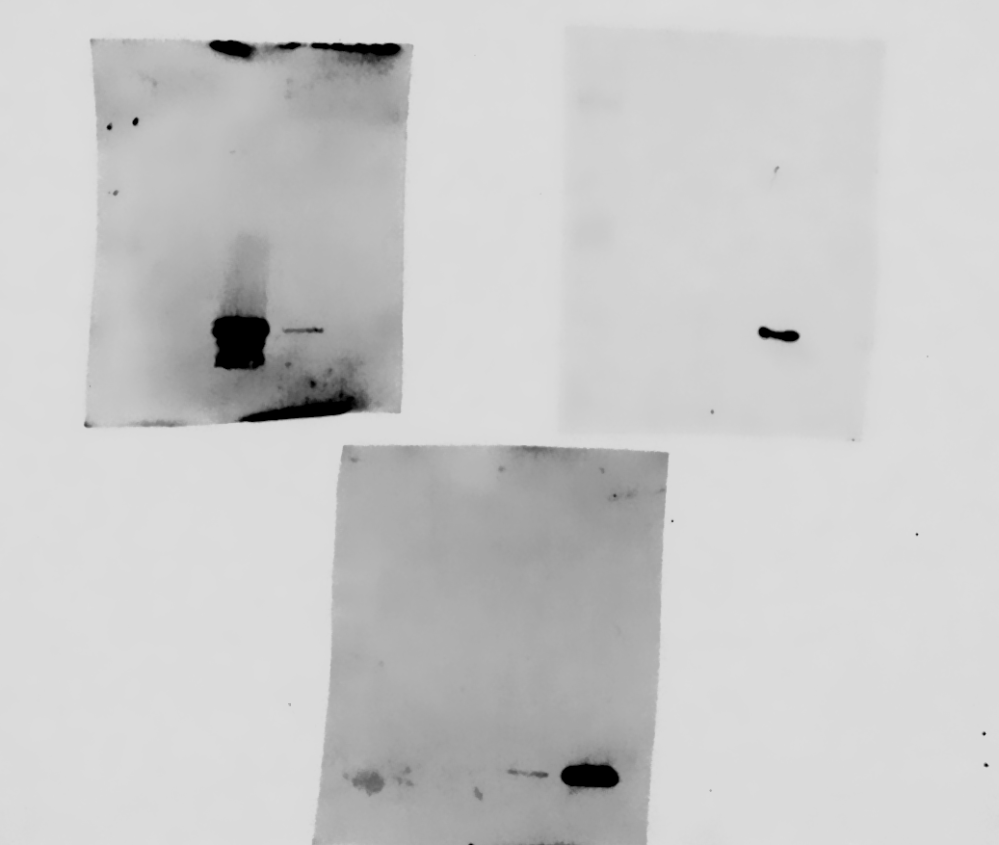

Supplement: Figure 2—source data 1. [file elife-107451-fig2-data1.zip › Figure 2_Source Data/Fig2Eme2me3.png.png]

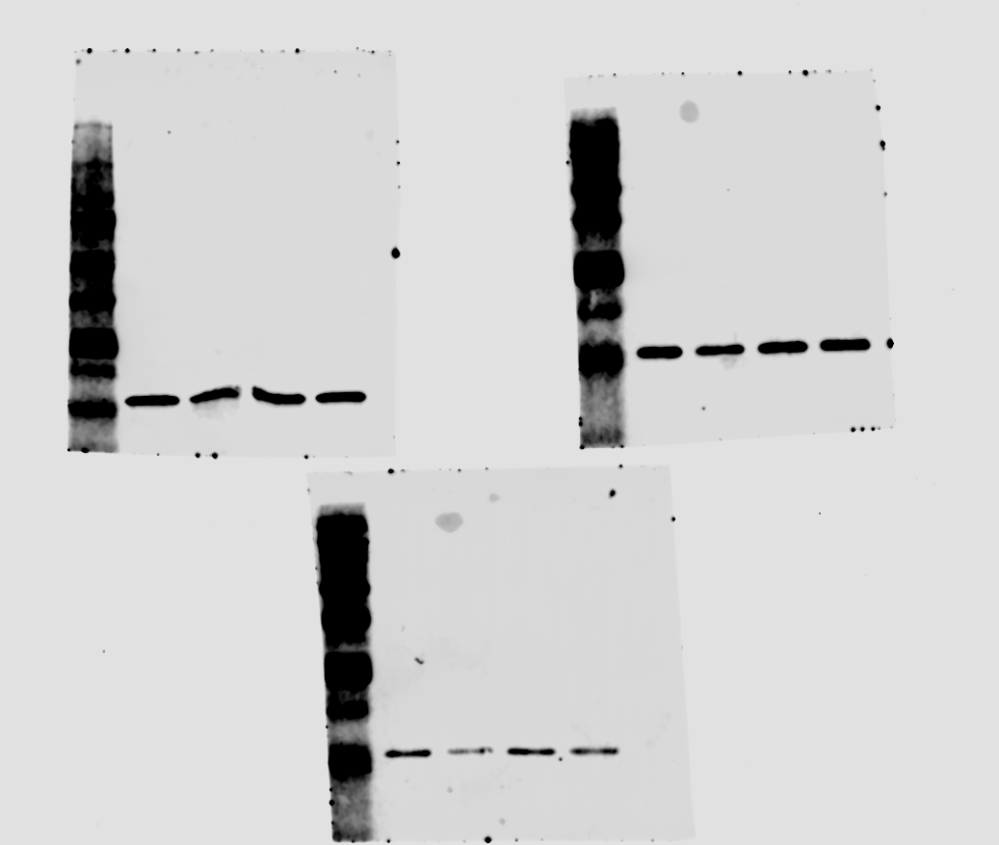

Supplement: Figure 2—source data 1. [file elife-107451-fig2-data1.zip › Figure 2_Source Data/Fig2FH3.png]

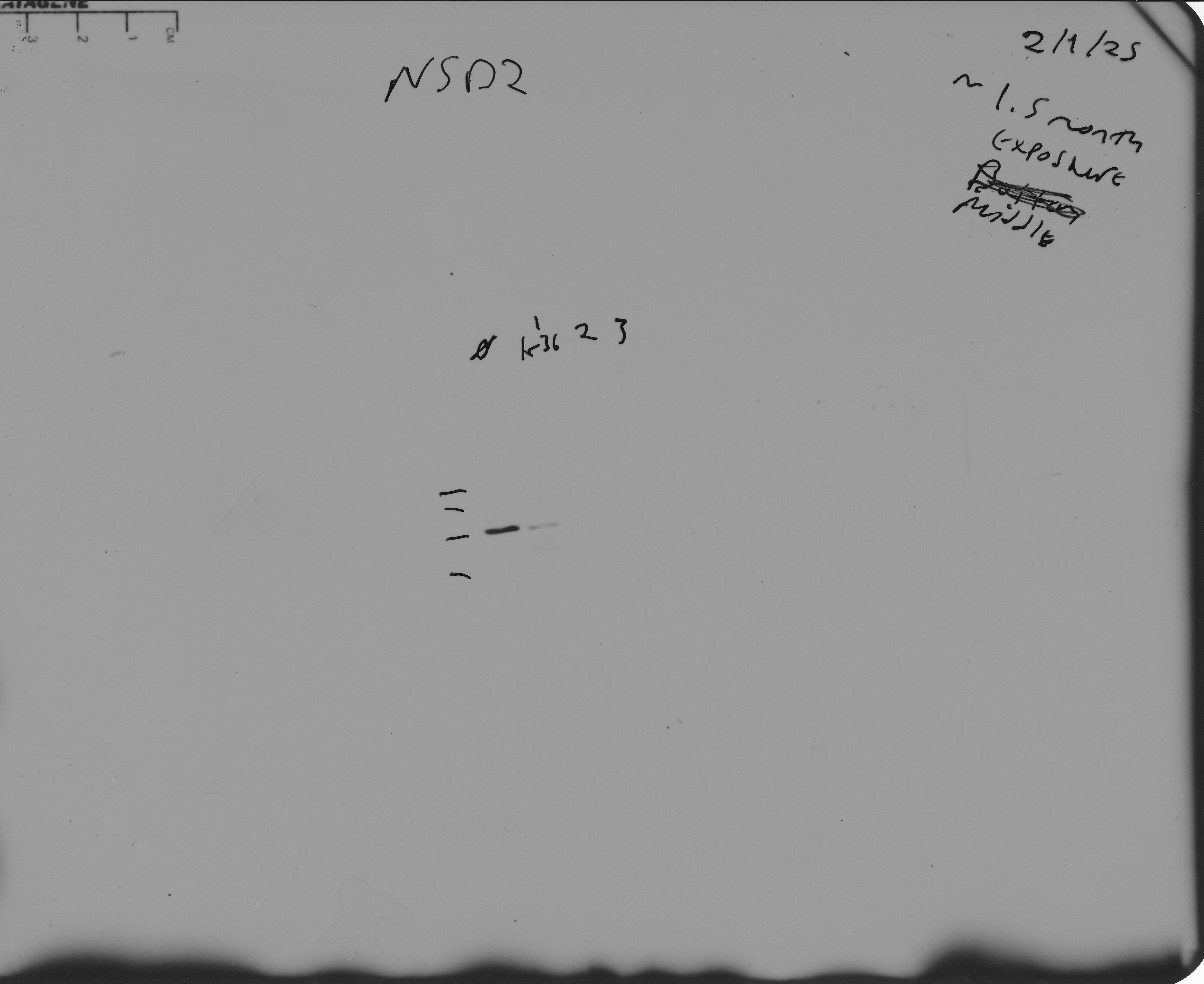

Supplement: Figure 2—source data 1. [file elife-107451-fig2-data1.zip › Figure 2_Source Data/Fig2D.jpg]

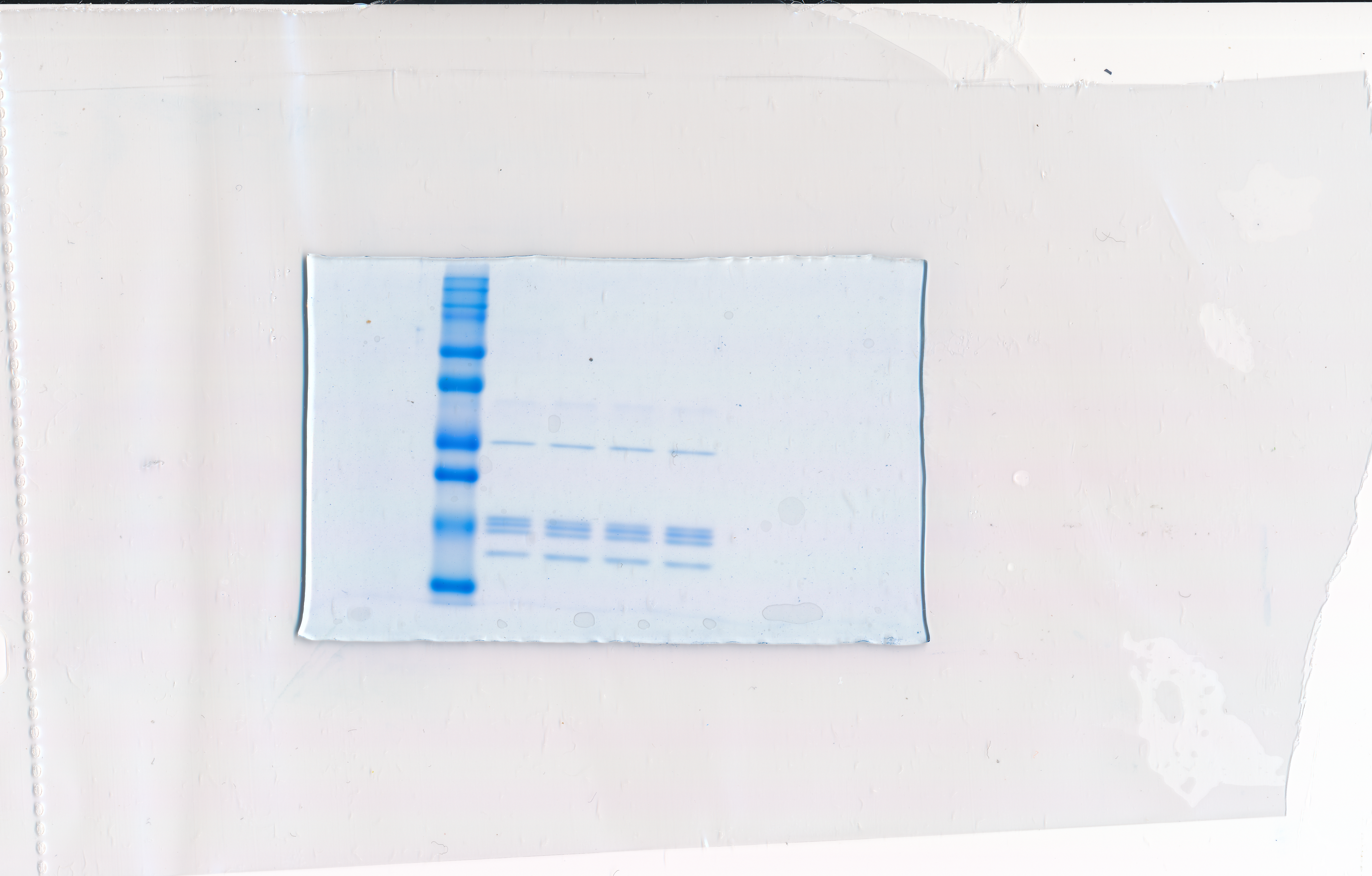

Supplement: Figure 2—source data 1. [file elife-107451-fig2-data1.zip › Figure 2_Source Data/Fig2Dk36me_C_121624_20241217_0001.tif]

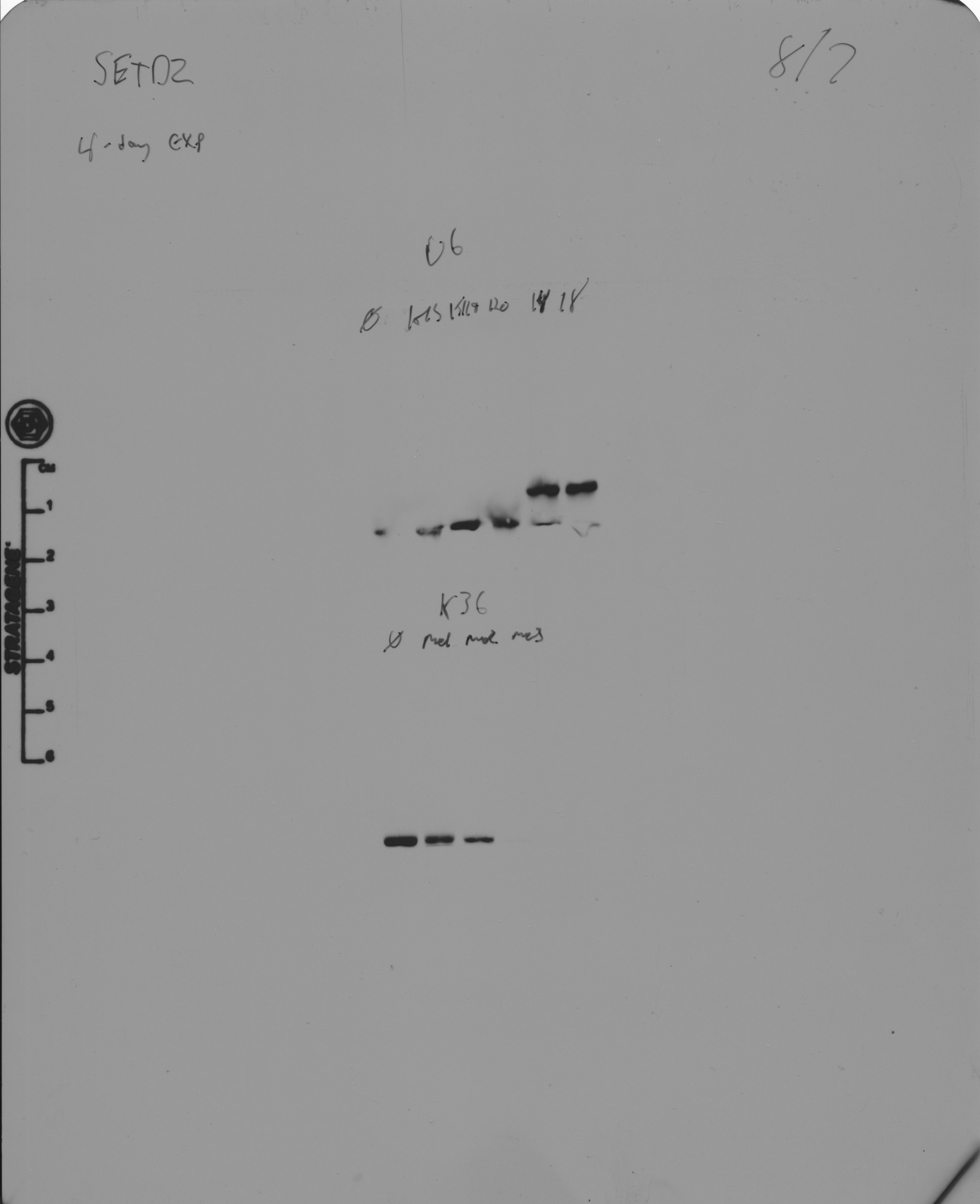

Supplement: Figure 2—source data 1. [file elife-107451-fig2-data1.zip › Figure 2_Source Data/Fig2B.jpg]

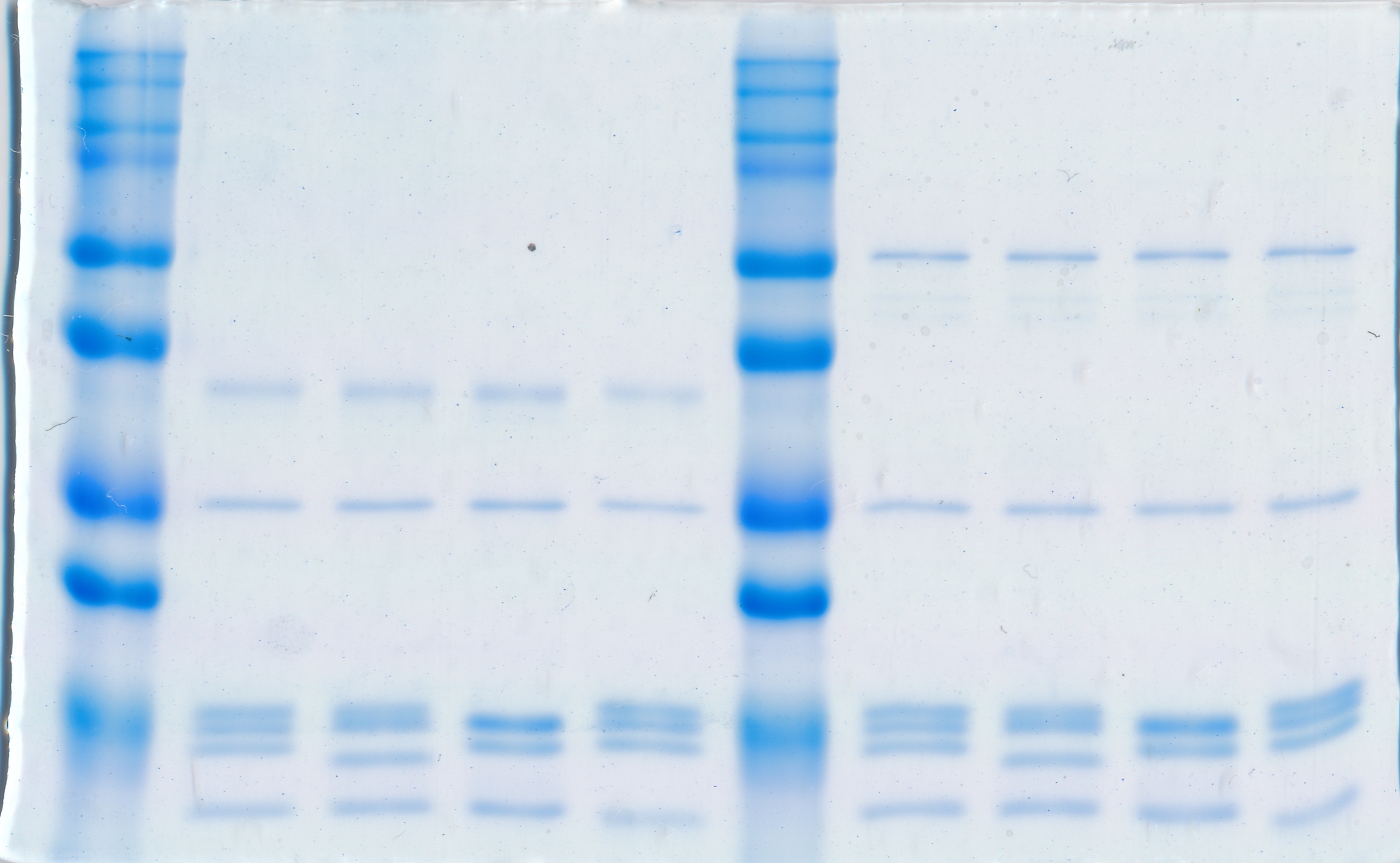

Supplement: Figure 3—source data 1. [file elife-107451-fig3-data1.zip › Figure 3_Source Data/Fig3E_C.jpg]

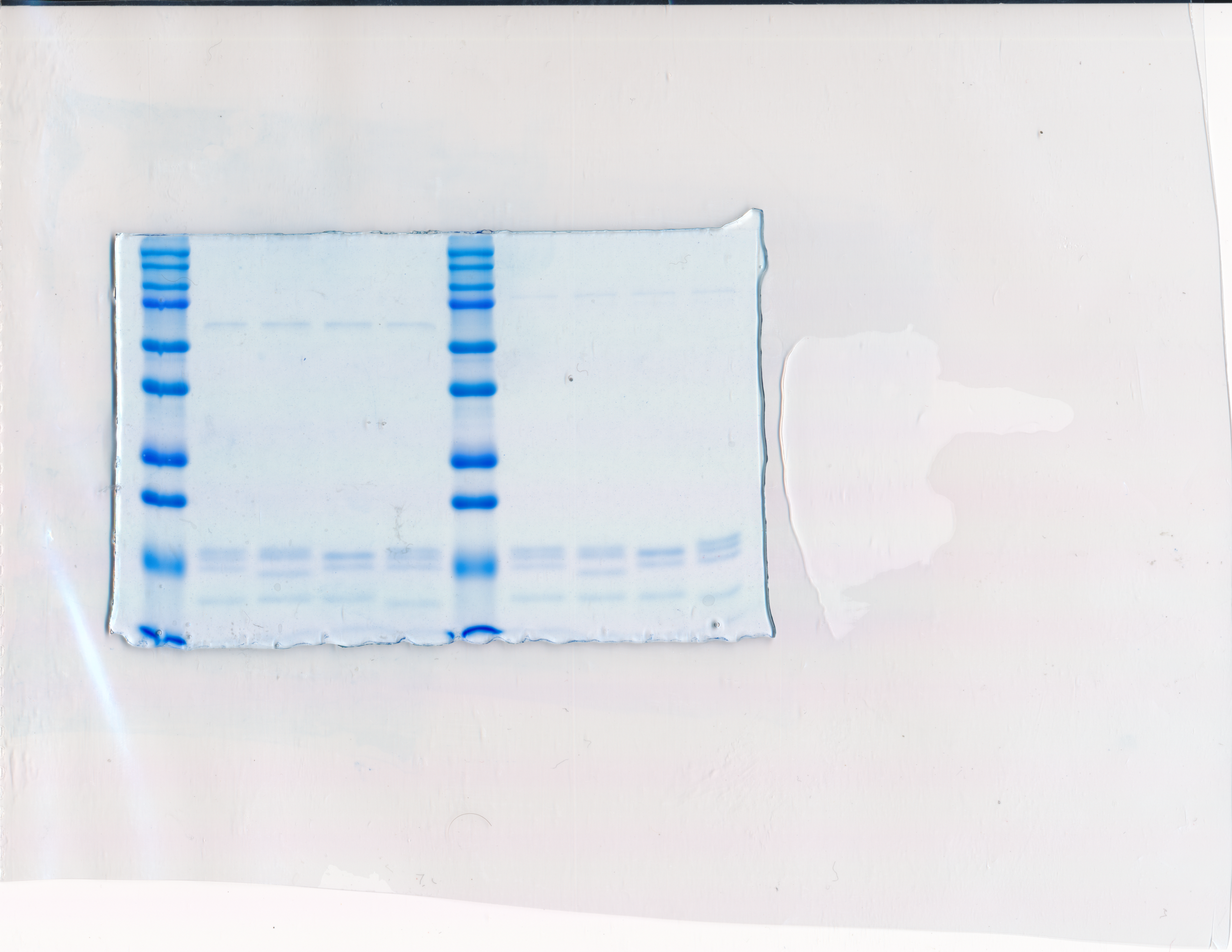

Supplement: Figure 3—source data 1. [file elife-107451-fig3-data1.zip › Figure 3_Source Data/Fig3B_C.tif]

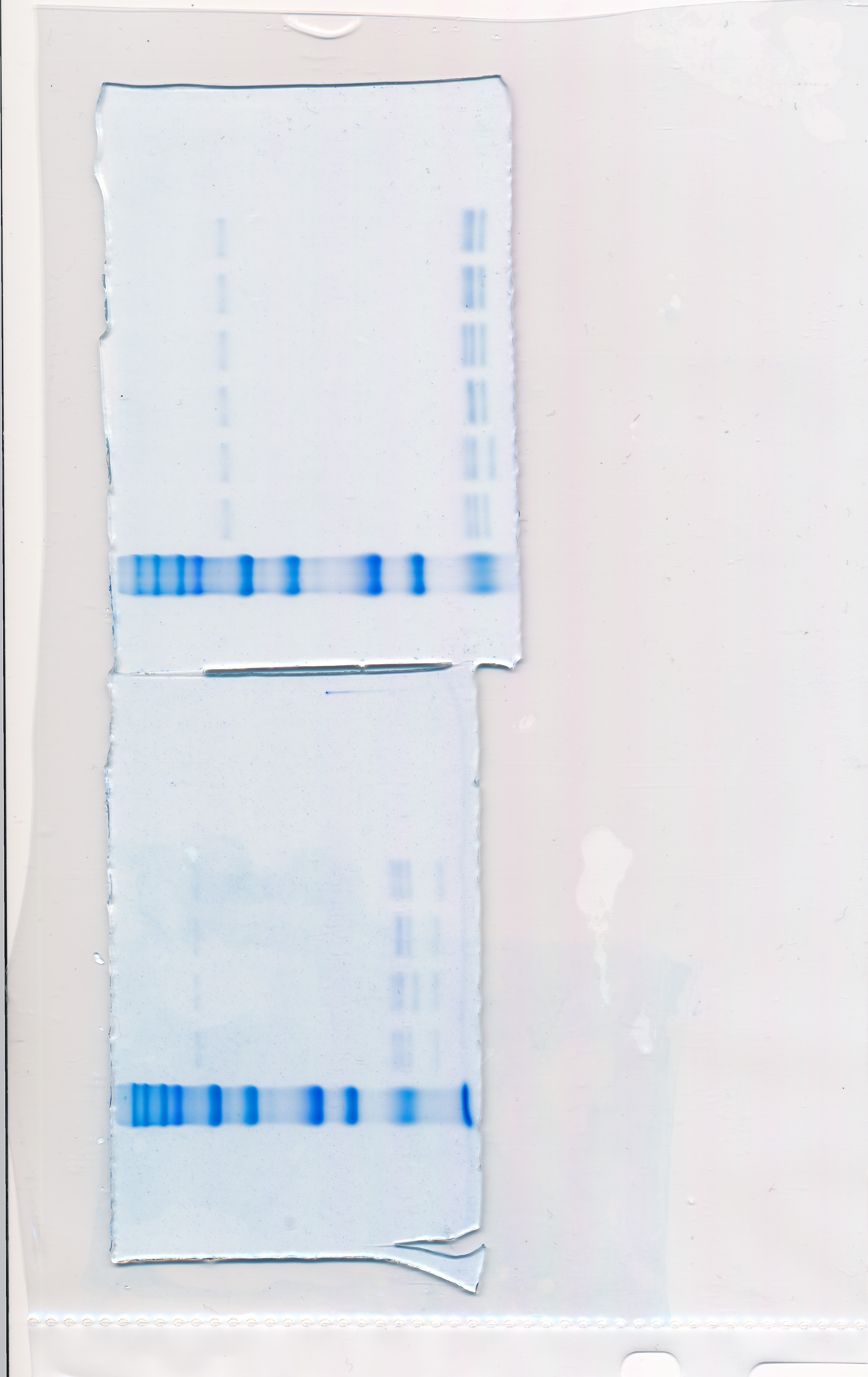

Supplement: Figure 3—source data 1. [file elife-107451-fig3-data1.zip › Figure 3_Source Data/Fig3D_C.jpg]

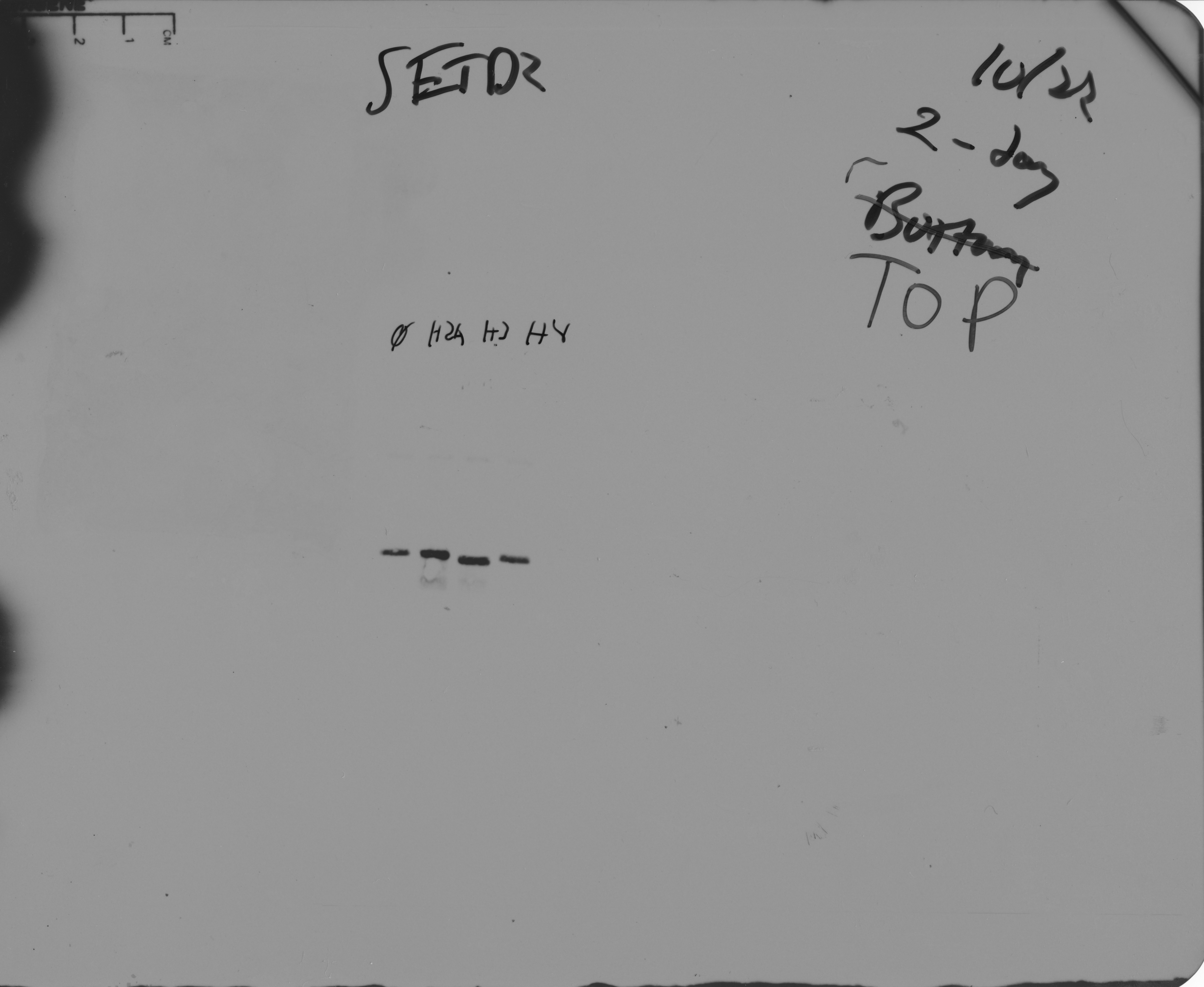

Supplement: Figure 3—source data 1. [file elife-107451-fig3-data1.zip › Figure 3_Source Data/Fig3B.jpg]

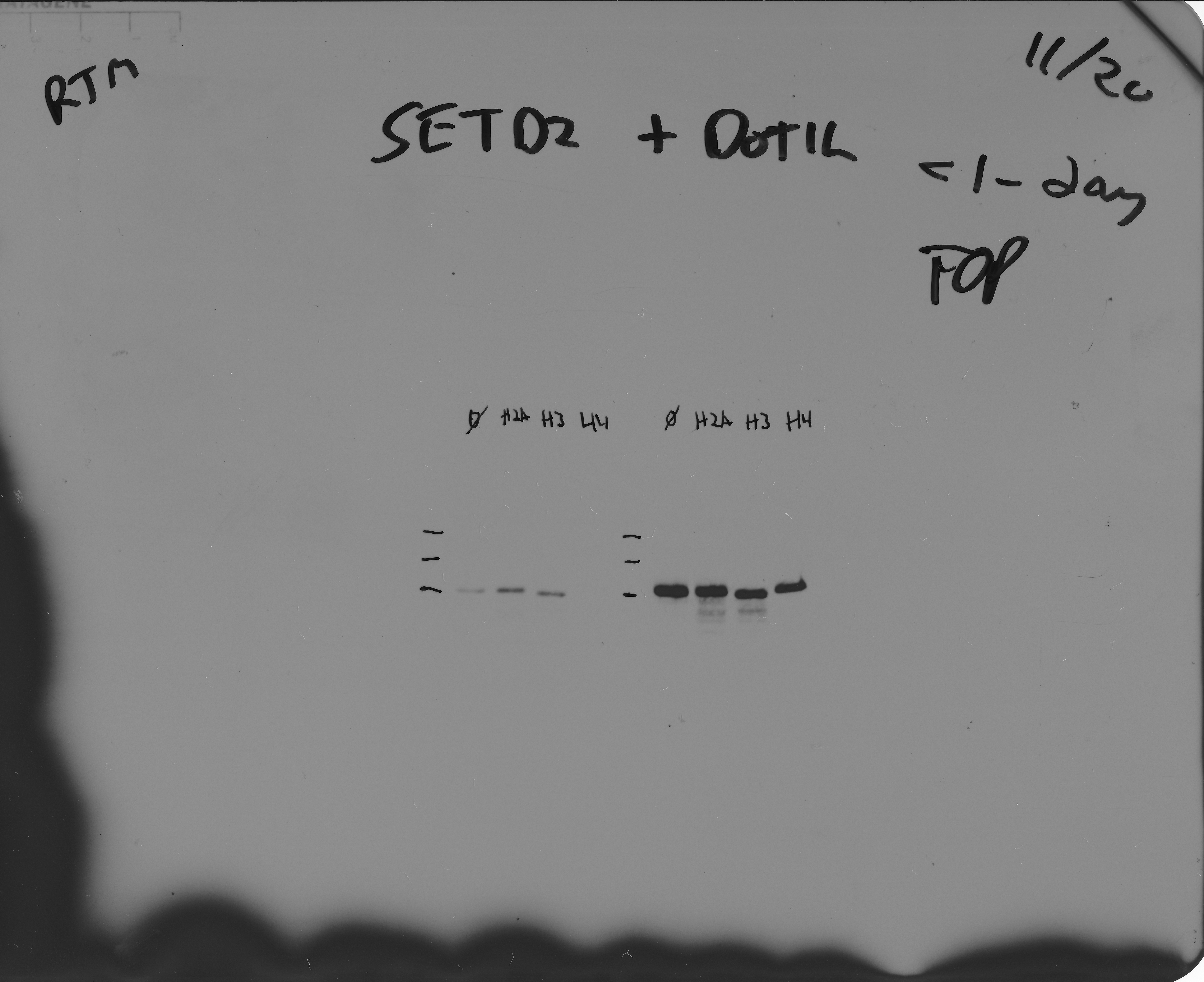

Supplement: Figure 3—source data 1. [file elife-107451-fig3-data1.zip › Figure 3_Source Data/Fig3E.jpg]

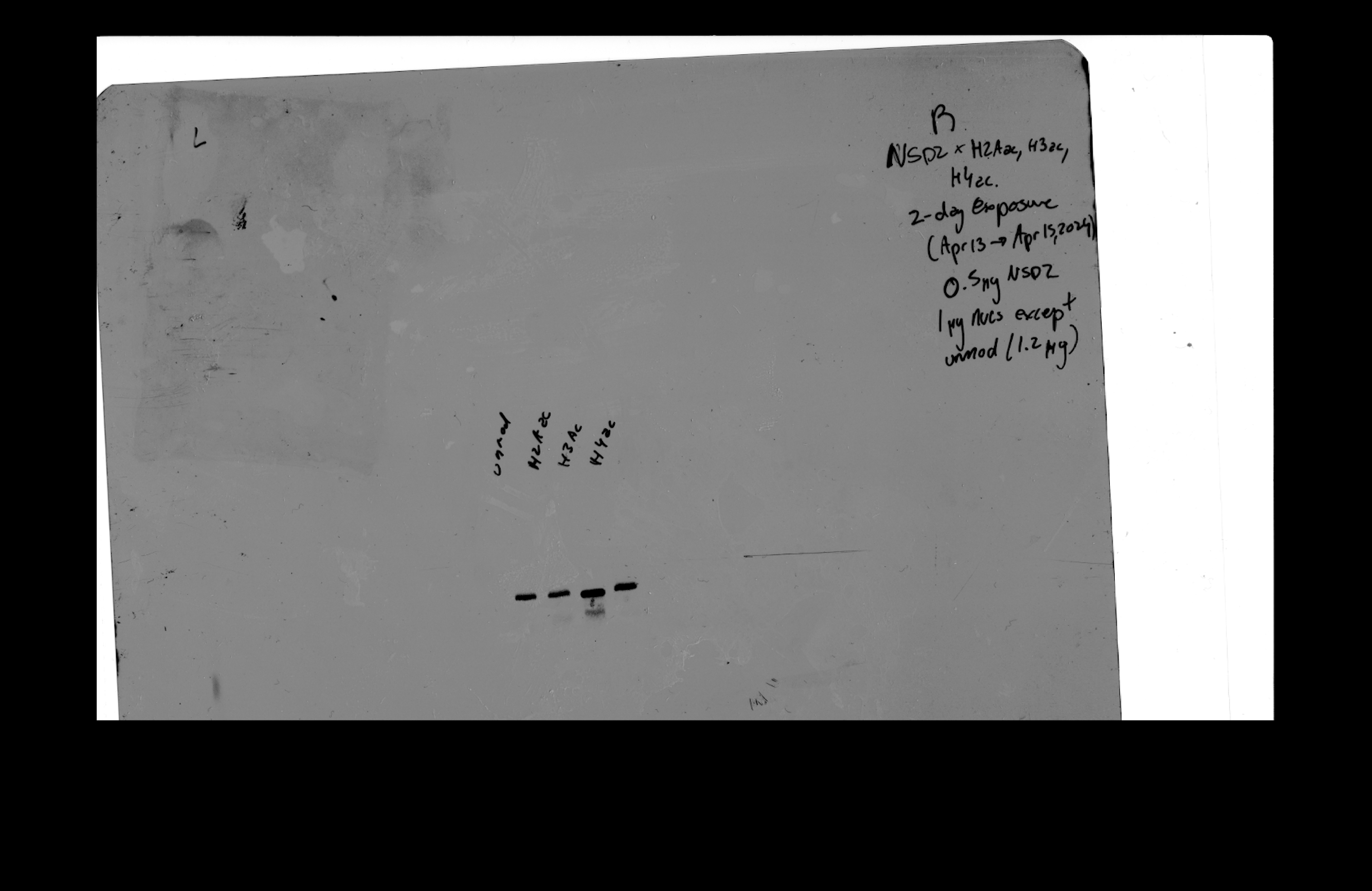

Supplement: Figure 3—source data 1. [file elife-107451-fig3-data1.zip › Figure 3_Source Data/Fig3D.png]

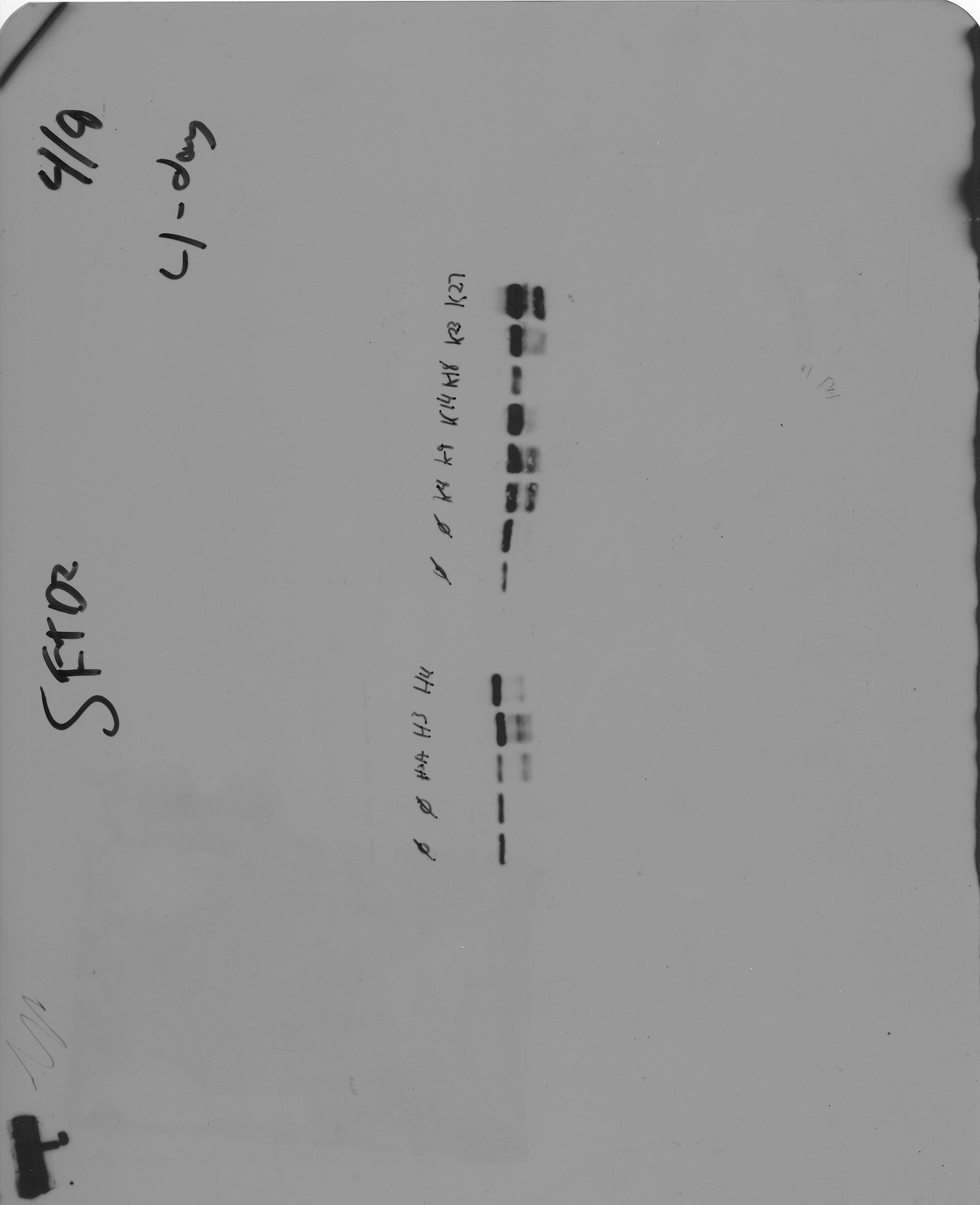

Supplement: Figure 4—source data 1. [file elife-107451-fig4-data1.zip › Figure 4_Source Data/Fig4B.jpg]

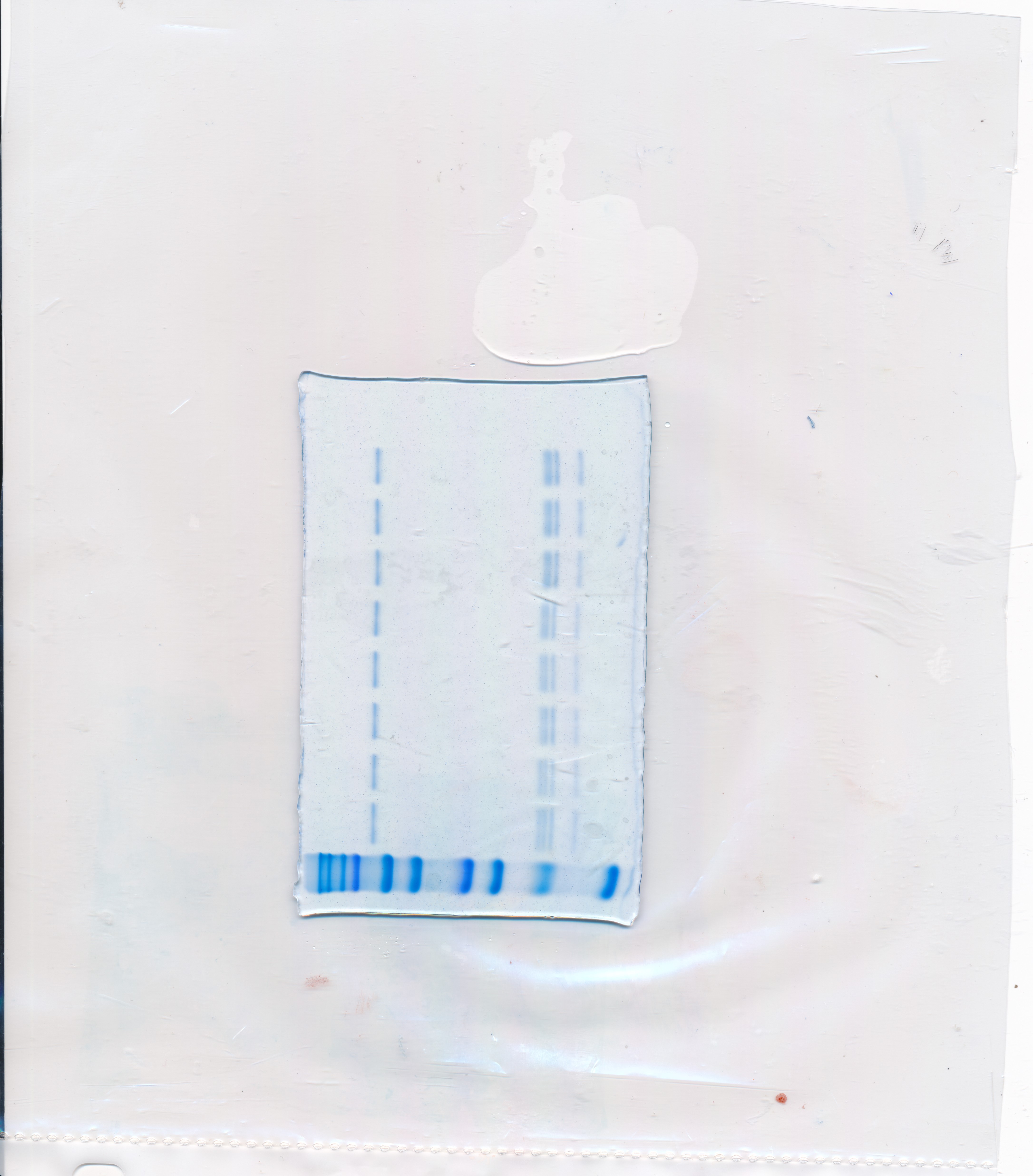

Supplement: Figure 4—source data 1. [file elife-107451-fig4-data1.zip › Figure 4_Source Data/Fig4B_C.jpg]

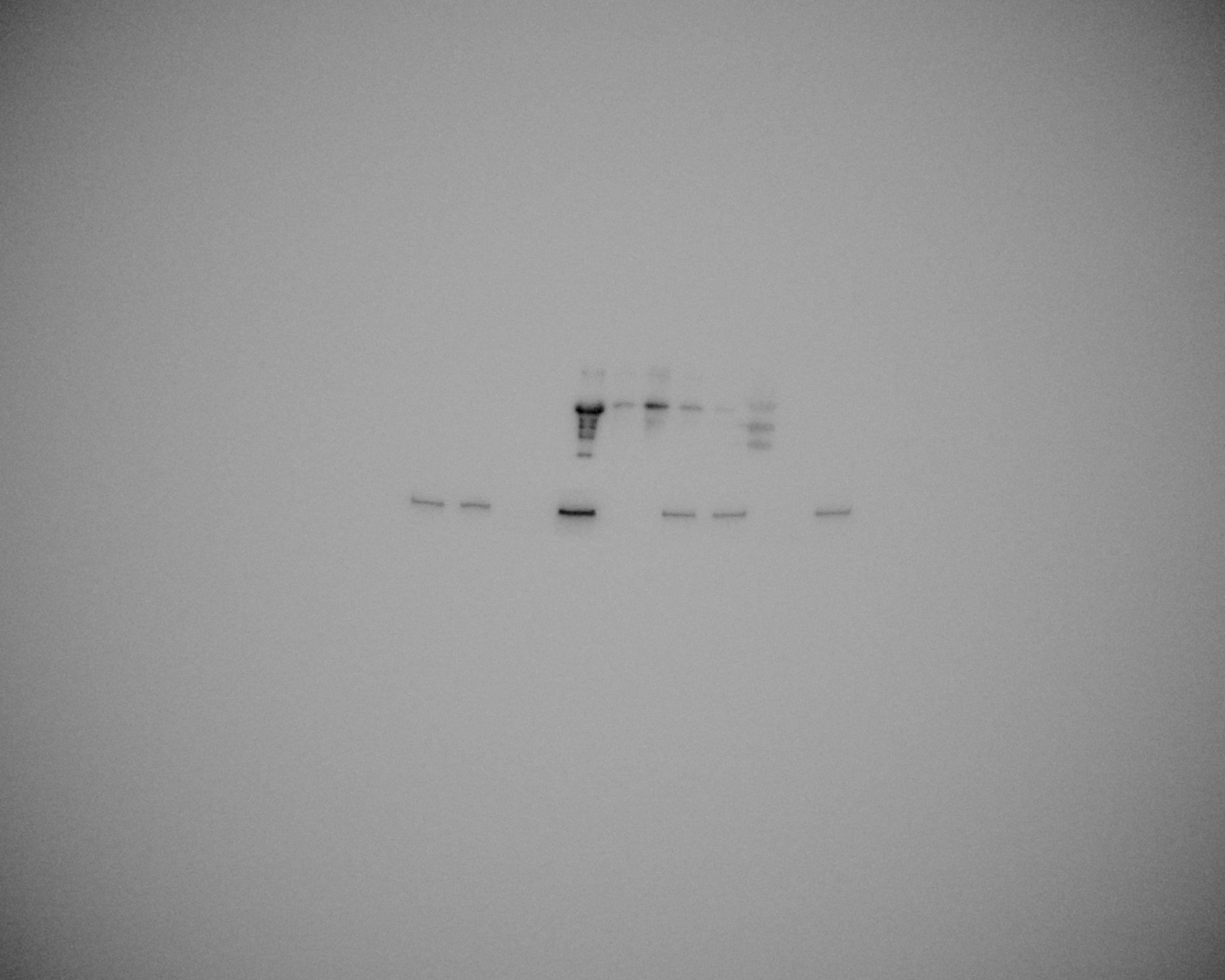

Supplement: Figure 4—source data 1. [file elife-107451-fig4-data1.zip › Figure 4_Source Data/Fig4D_1.tif]

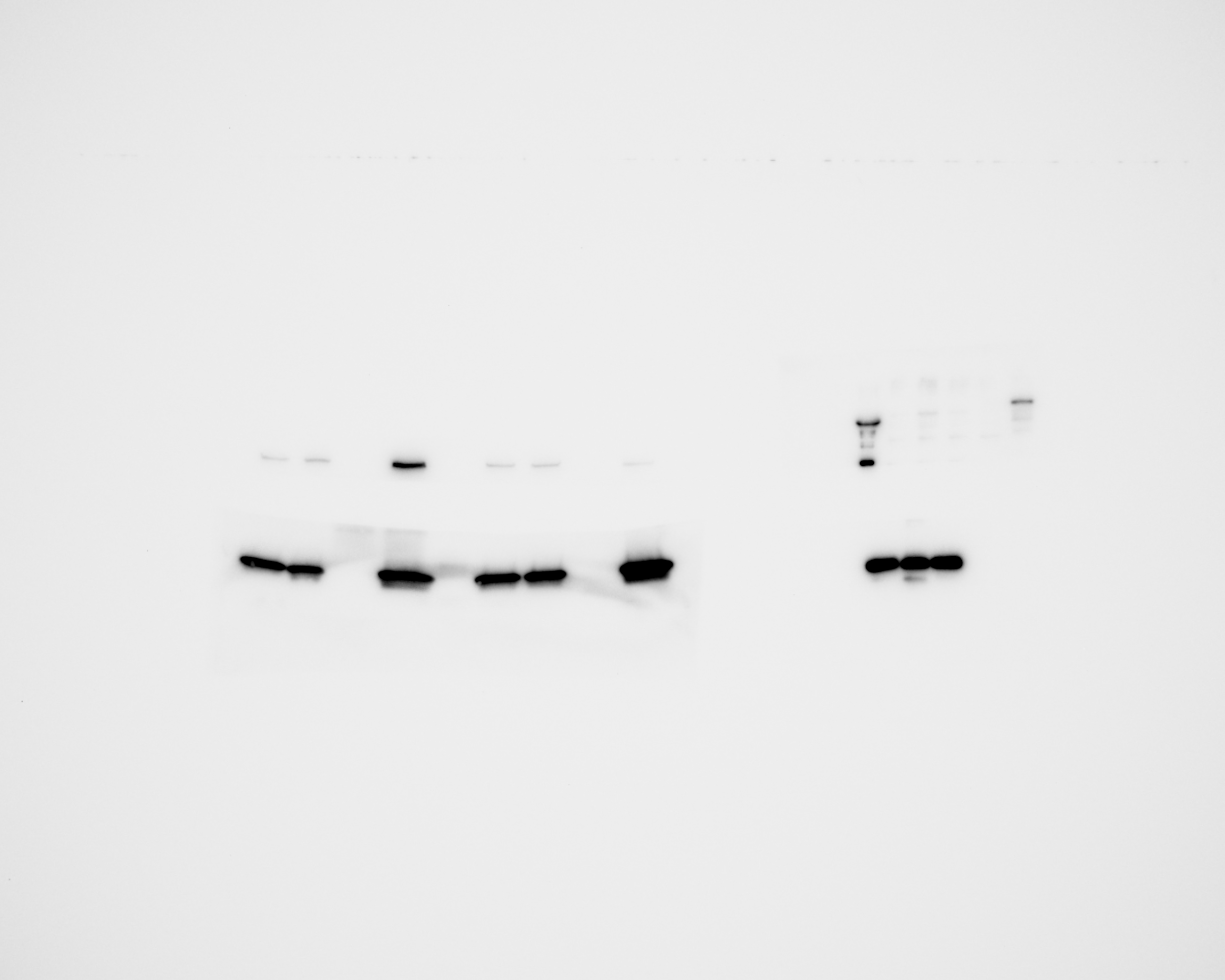

Supplement: Figure 4—source data 1. [file elife-107451-fig4-data1.zip › Figure 4_Source Data/Fig4D_2.tif]

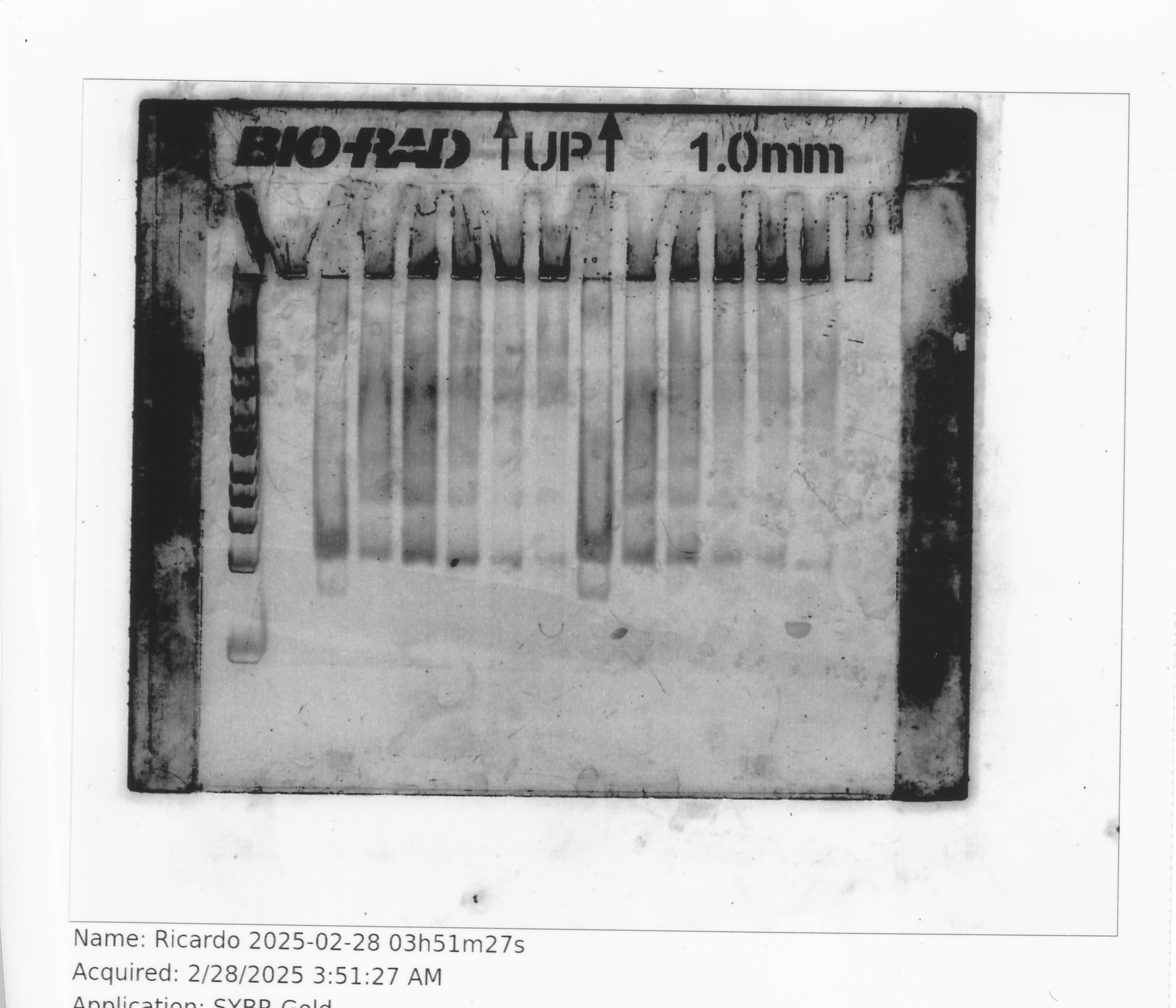

Supplement: Figure 4—source data 1. [file elife-107451-fig4-data1.zip › Figure 4_Source Data/Fig4E.tif]

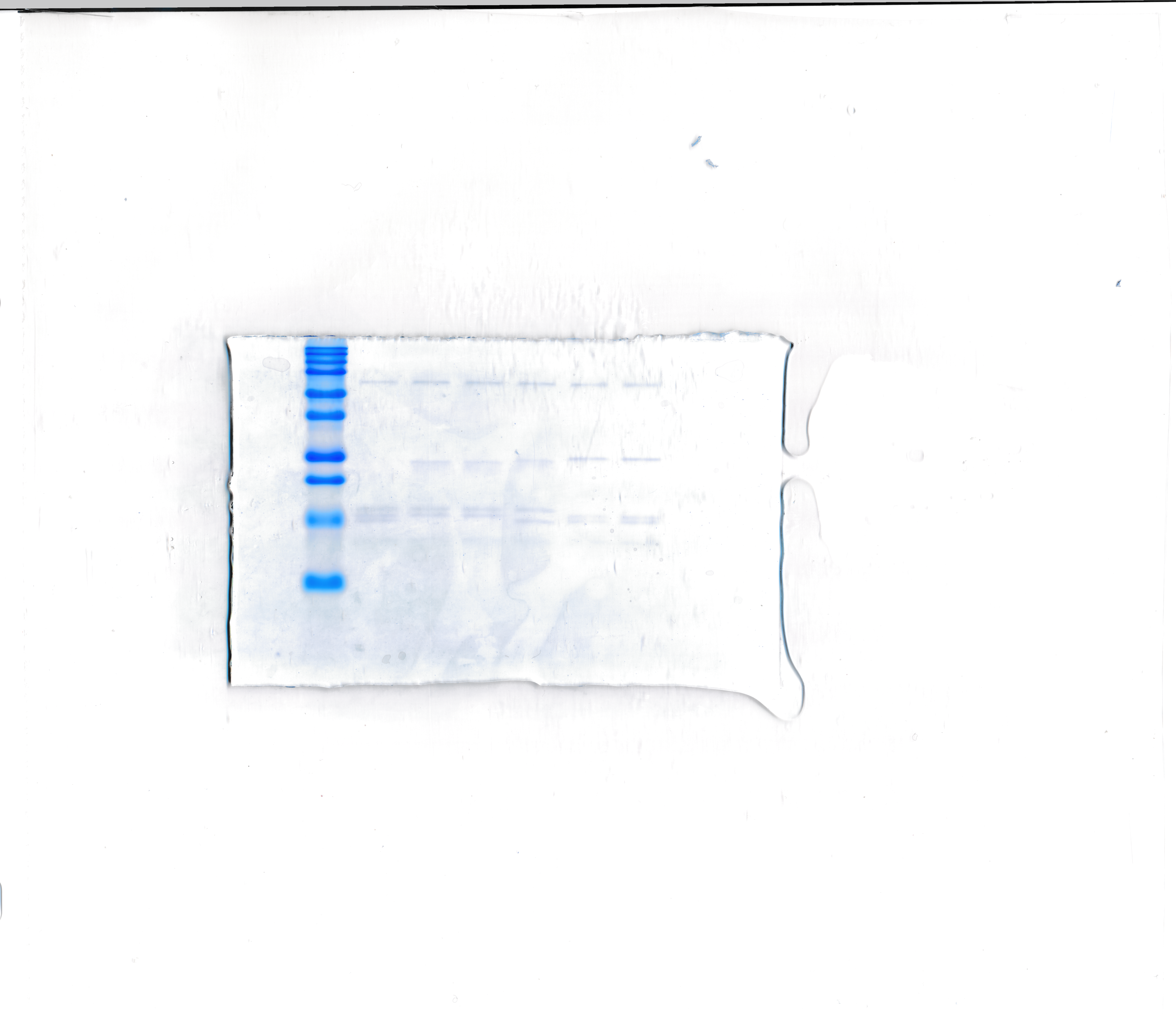

Supplement: Figure 5—source data 1. [file elife-107451-fig5-data1.zip › Figure 5_Source Data/Fig5B_C.png]

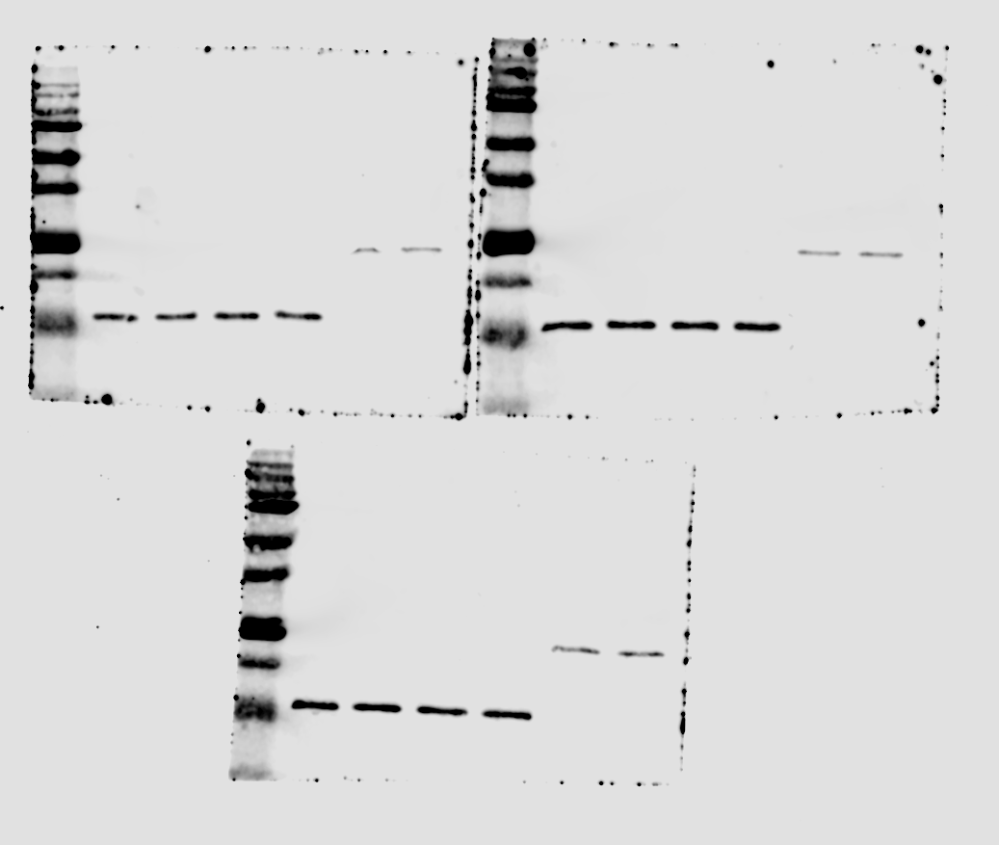

Supplement: Figure 5—source data 1. [file elife-107451-fig5-data1.zip › Figure 5_Source Data/Fig5CH3.png.png]

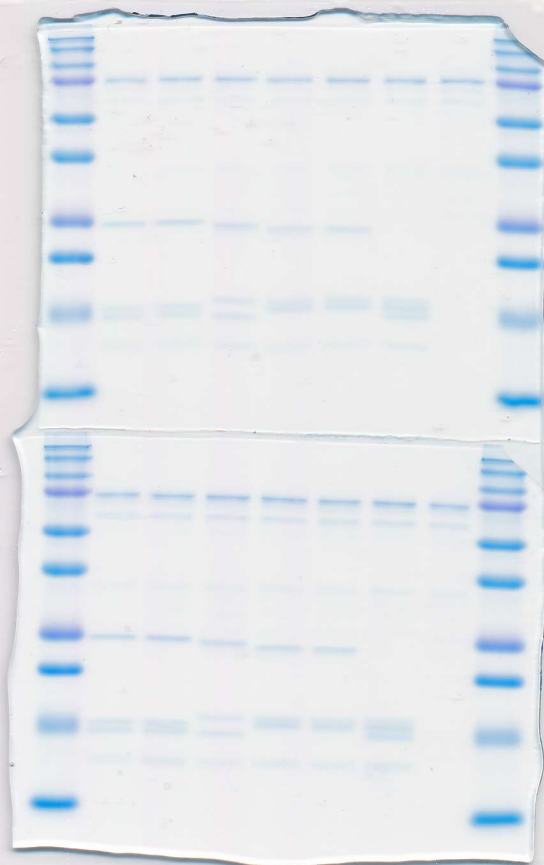

Supplement: Figure 5—source data 1. [file elife-107451-fig5-data1.zip › Figure 5_Source Data/Fig5E_C.pdf]

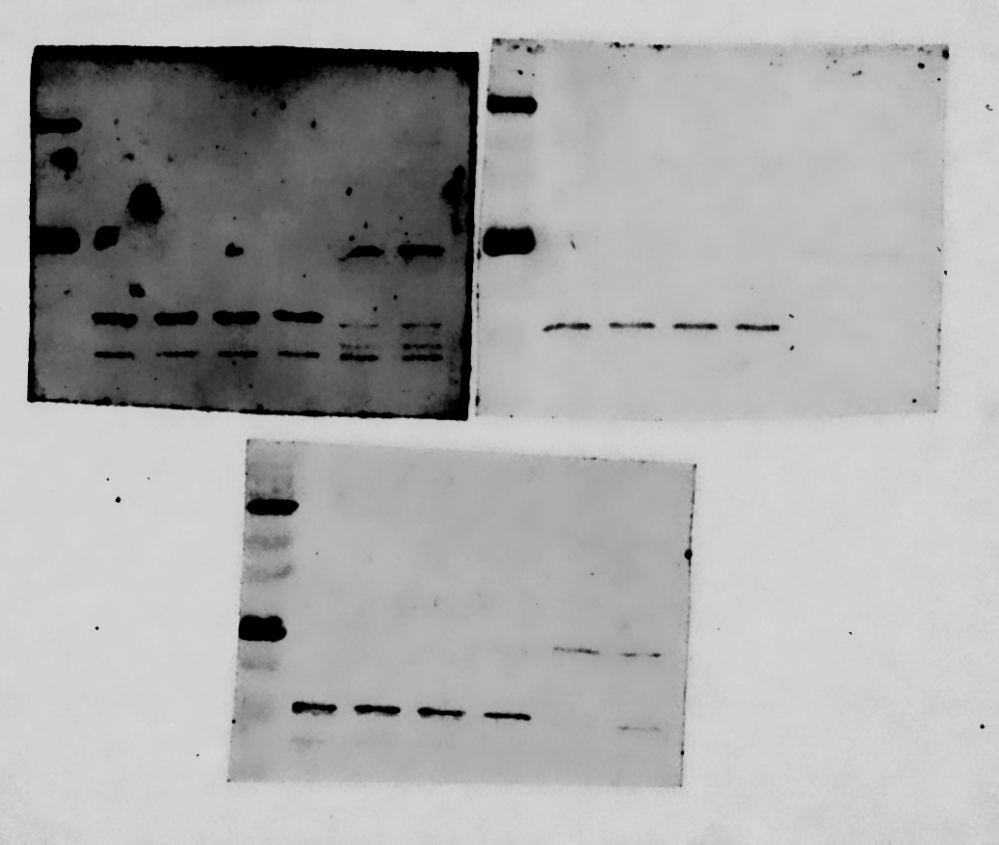

Supplement: Figure 5—source data 1. [file elife-107451-fig5-data1.zip › Figure 5_Source Data/Fig5Cme2me3.png.png]

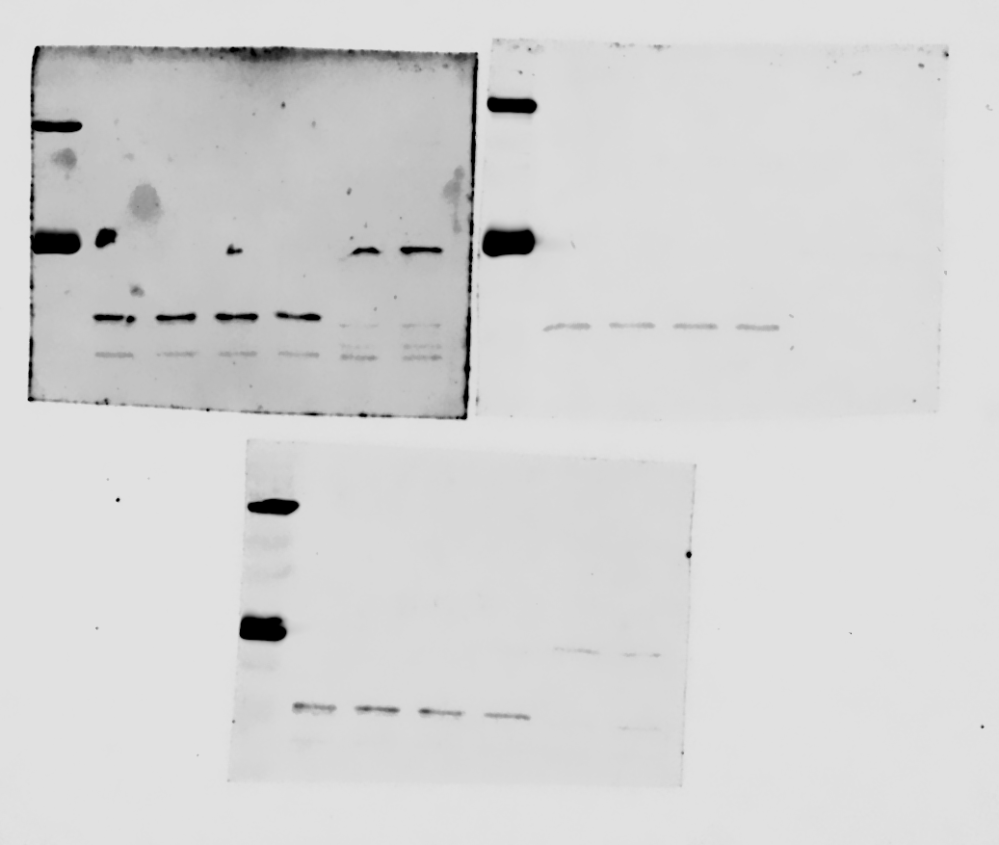

Supplement: Figure 5—source data 1. [file elife-107451-fig5-data1.zip › Figure 5_Source Data/Fig5Cme1.png.png]

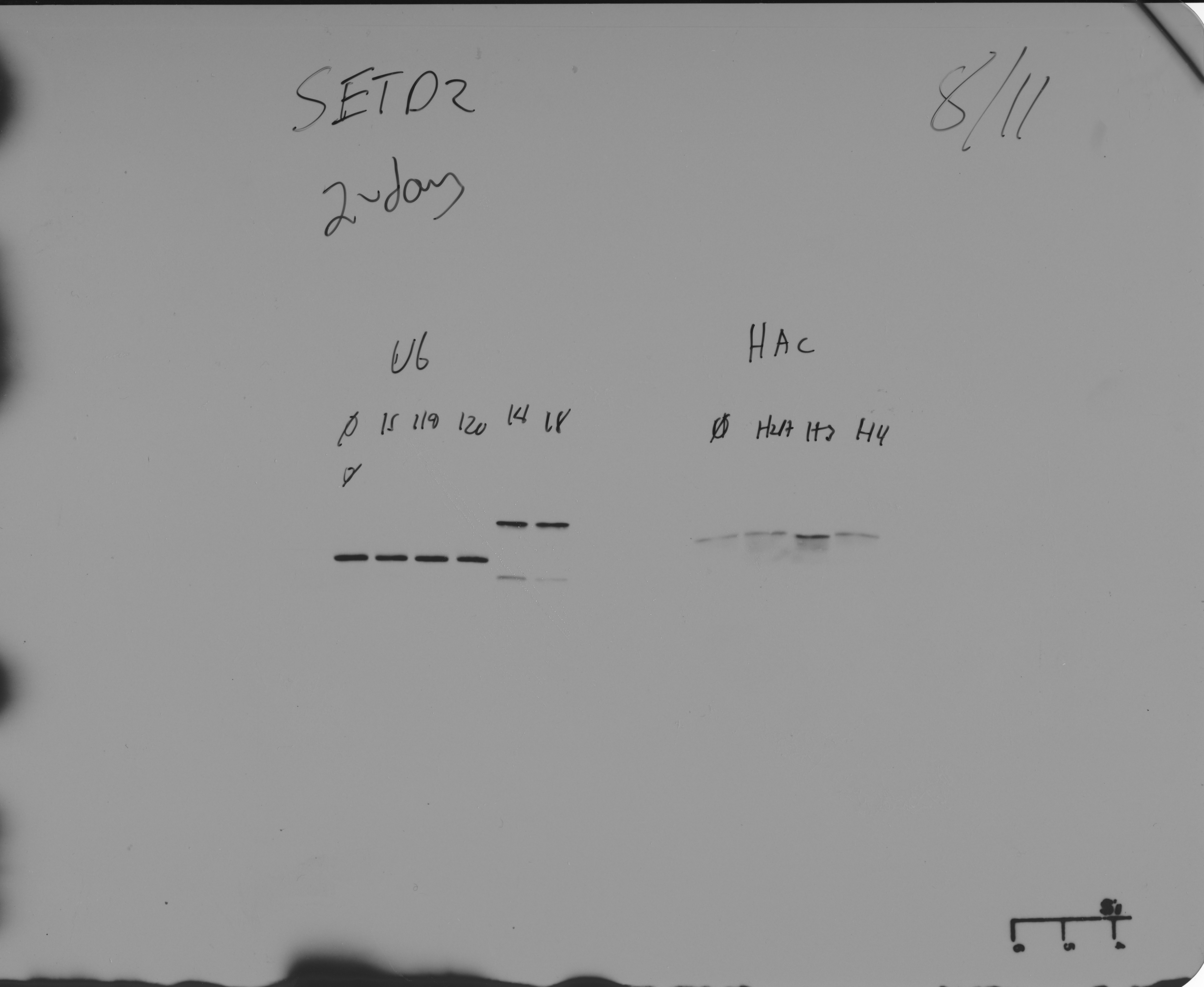

Supplement: Figure 5—source data 1. [file elife-107451-fig5-data1.zip › Figure 5_Source Data/Fig5B.png]
