# Supplementary material for: SETD2 suppresses tumorigenesis in a KRASG12C-driven lung cancer model, and its catalytic activity is regulated by histone acetylation
Source: eLife. 2025 Sep 15;14:RP107451. doi: 10.7554/eLife.107451 (PMC12435893; doi:10.7554/eLife.107451)
Supplement: Figure 2—source data 2. [file elife-107451-fig2-data2.zip › Figure 2 SourceData_Labeled.docx]

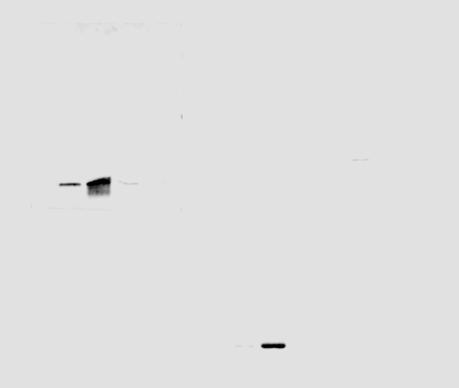

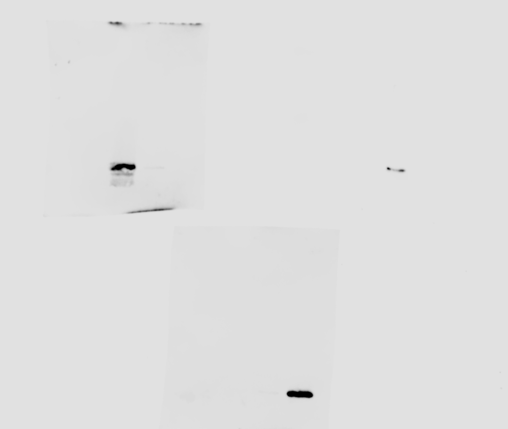

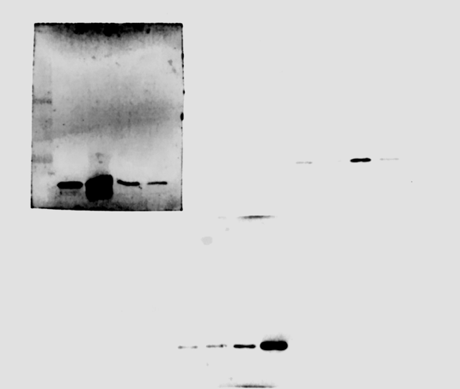

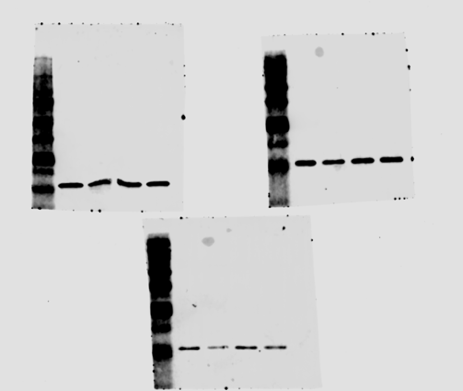

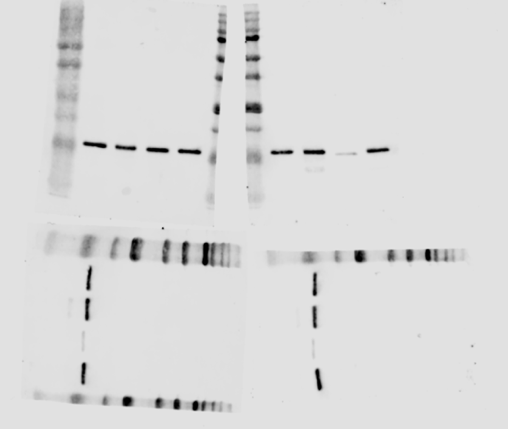

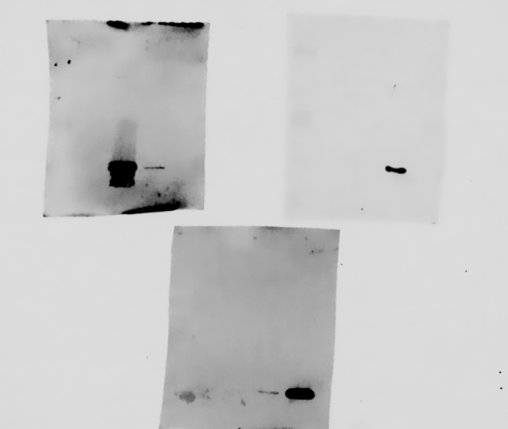

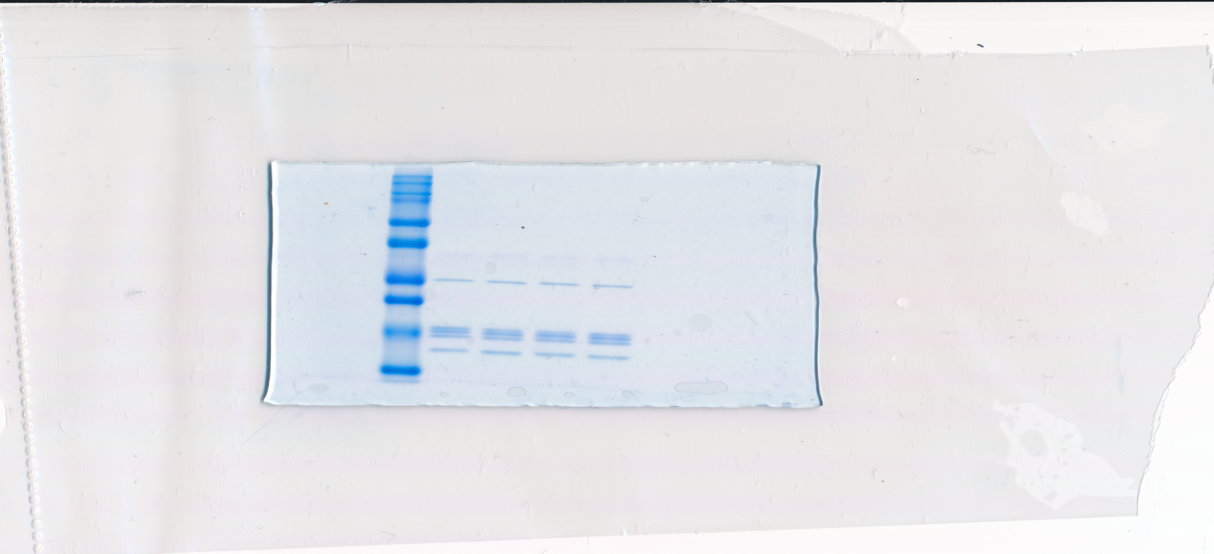

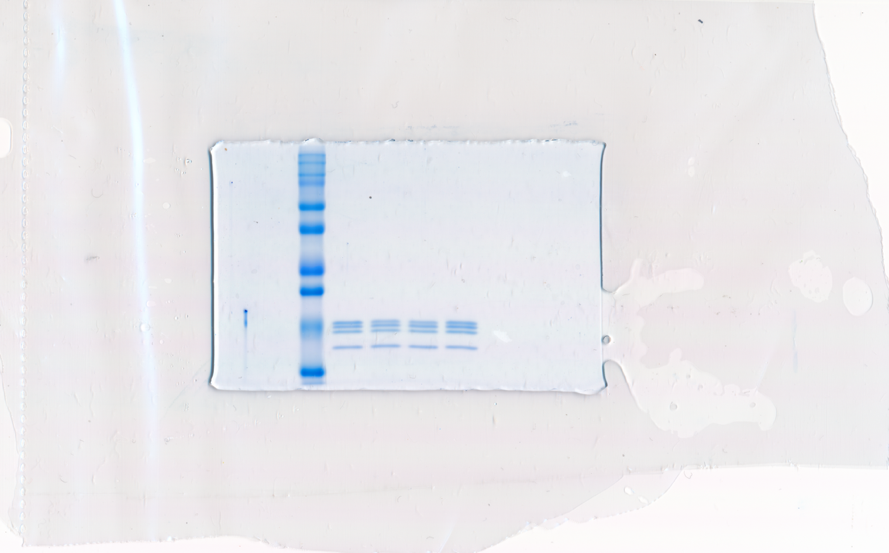

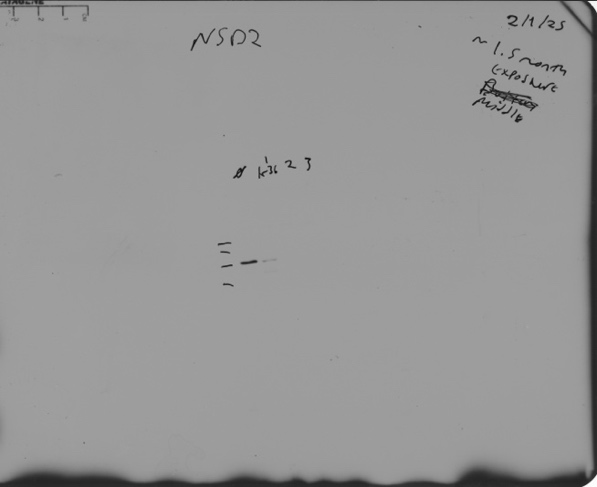

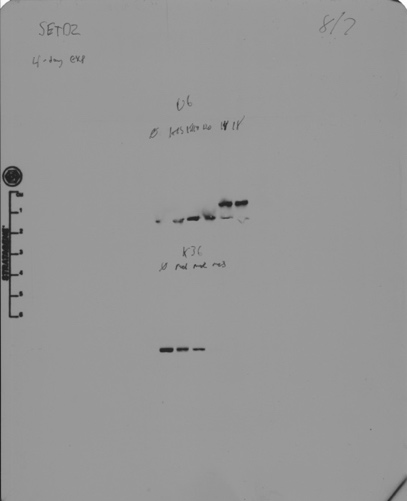


15kDa –

15kDa –

15kDa –

15kDa –

15kDa –

15kDa –

15kDa –

15kDa –

15kDa –

**Figure 2, Source Data.** Original films, gels, and membranes corresponding to Figure 2, panel B, D, E, and F. Precision Plus molecular weight markers were used. Corresponding panels B and D display the methylation activity of SETD2 and NSD2 respectively with the loading control gels for the nucleosomes(relevant bands have been marked). Membranes for Panels E and F have also been marked to identify the relevant bands.

SETD2 – H3K36me1

SETD2 – H3K36me2/ me3 control

SETD2 – H3

SETD2 – H3K36me2/ me3

15kDa –

20kDa –

10kDa –

SETD2 – H3 control

SETD2 – H3K36me1 control

15kDa –

20kDa –

10kDa –

10kDa –

15kDa –

20kDa –

SETD2 - K36 Methylation

NSD2 - K36 Methylation
