# Supplementary material for: SETD2 suppresses tumorigenesis in a KRASG12C-driven lung cancer model, and its catalytic activity is regulated by histone acetylation
Source: eLife. 2025 Sep 15;14:RP107451. doi: 10.7554/eLife.107451 (PMC12435893; doi:10.7554/eLife.107451)
Supplement: Figure 3—source data 2. [file elife-107451-fig3-data2.zip › Figure 3 SourceData_Labeled.docx]

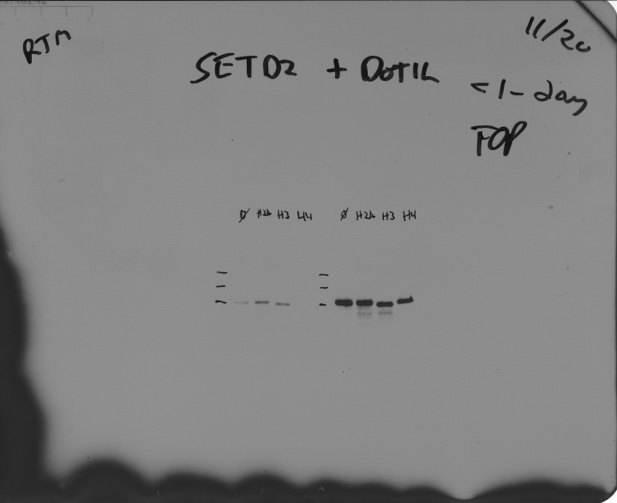

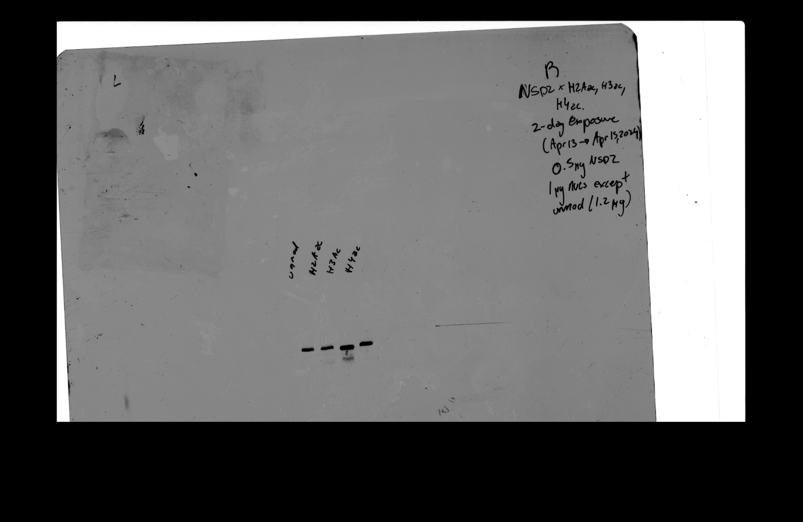

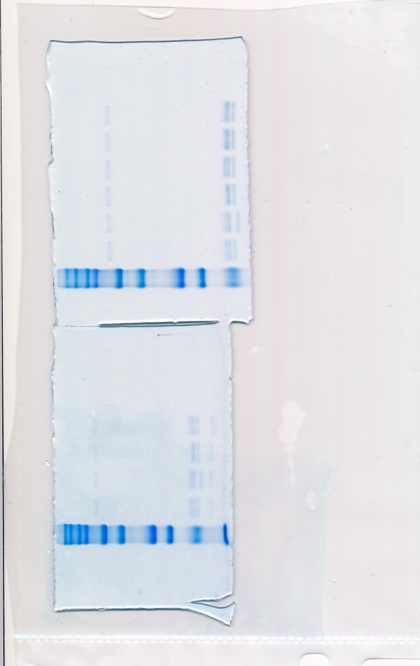

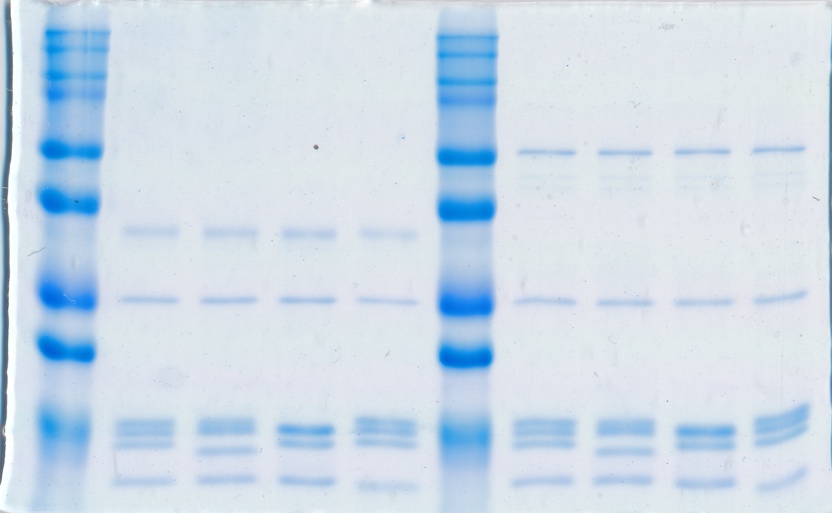

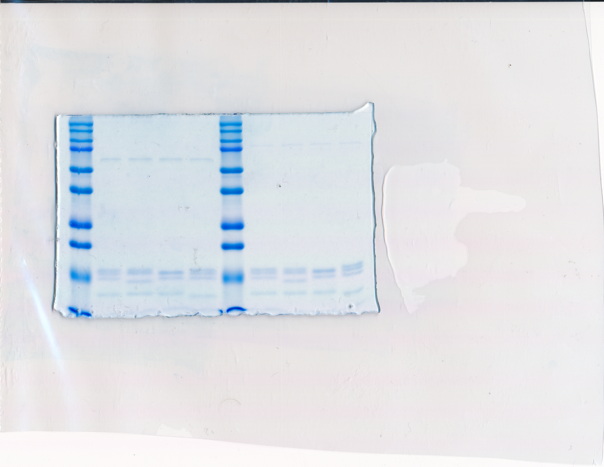

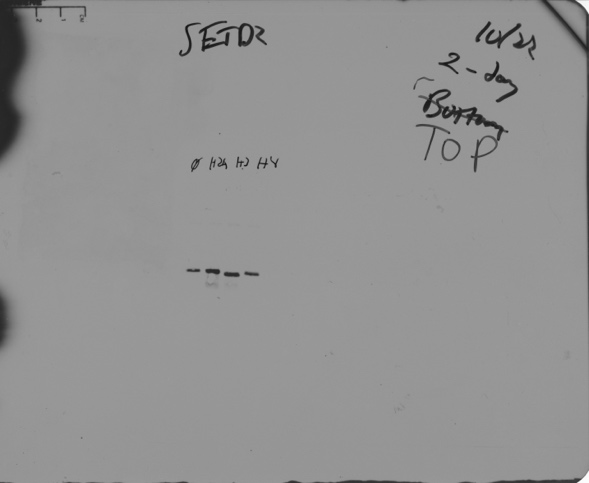


**Figure 3, Source Data.** Original films and gels corresponding to Figure 3, panel B, D, and E. Precision Plus molecular weight markers were used. Corresponding panels B, D, and E display the methylation activity of SETD2, NSD2, and DOT1L respectively, with the loading control gels for the nuclesomes(relevant bands have been marked).

15kDa –

DOT1L – K79 Methylation

15kDa –

20kDa –

15kDa –

15kDa –

20kDa –

10kDa –

10kDa –

15kDa –

20kDa –

15kDa –

NSD2 - K36 Methylation

SETD2 - K36 Methylation
