# Supplementary material for: SETD2 suppresses tumorigenesis in a KRASG12C-driven lung cancer model, and its catalytic activity is regulated by histone acetylation
Source: eLife. 2025 Sep 15;14:RP107451. doi: 10.7554/eLife.107451 (PMC12435893; doi:10.7554/eLife.107451)
Supplement: Figure 4—source data 2. [file elife-107451-fig4-data2.zip › Figure 4 SourceData_Labeled.docx]

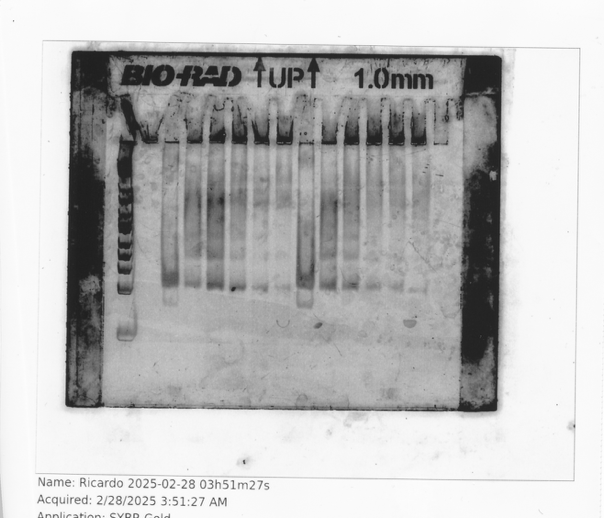

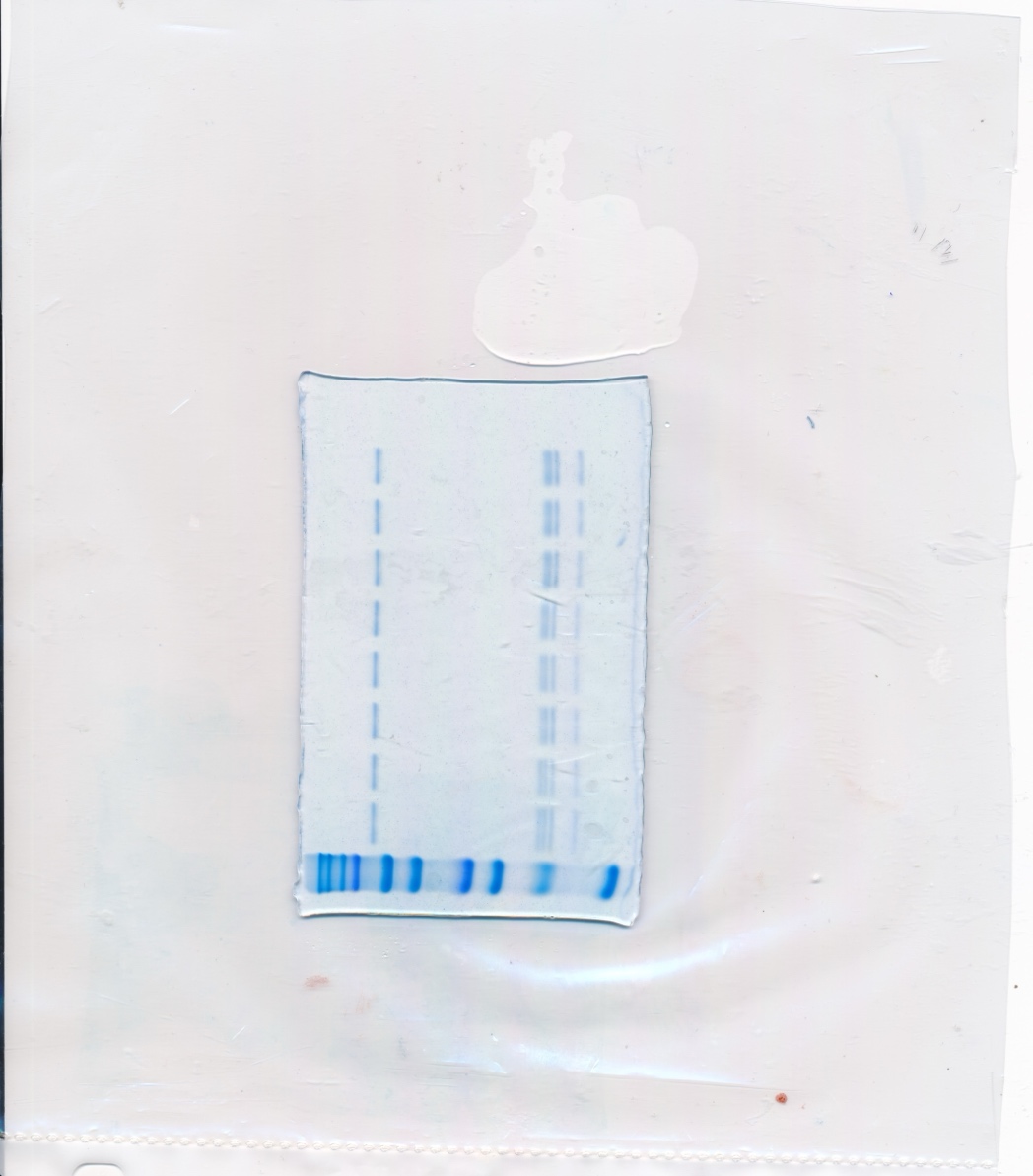

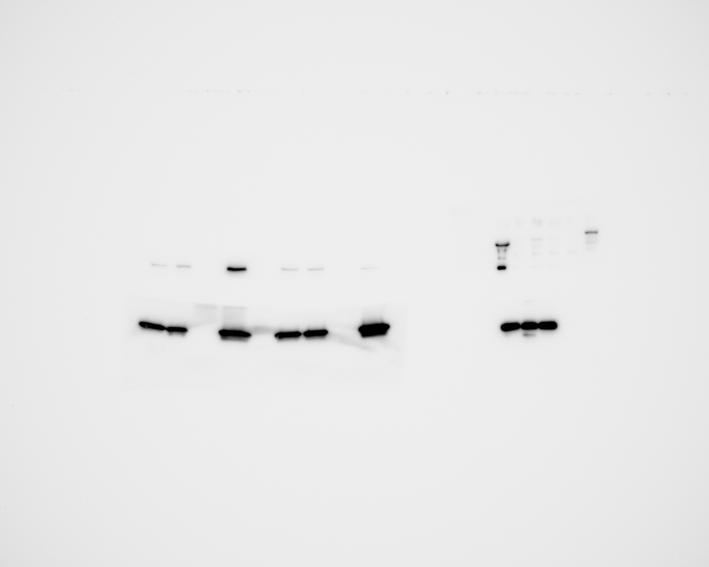

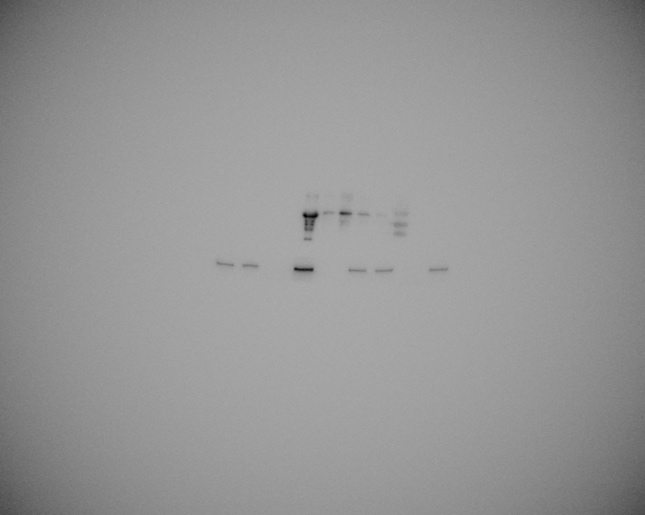

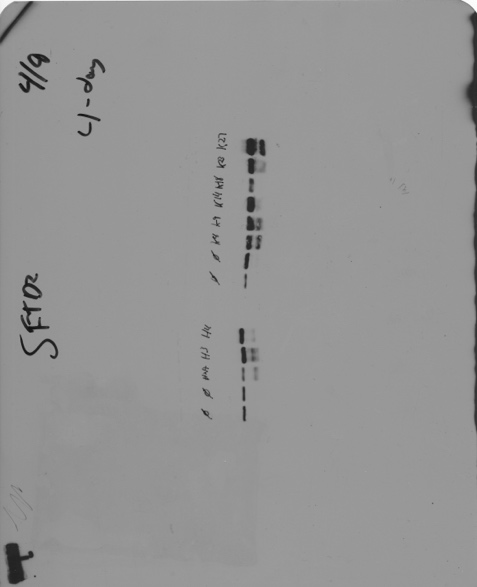


H3K27ac

H3

input

H3K27ac

input

H3

aSETD2

aH3

SETD2 – K27ac pulldown

200bp –

300bp –

15kDa –

20kDa –

10kDa –

15kDa –

**Figure 4, Source Data.** Original films and gels corresponding to Figure 4, panel B, D, and E. Precision Plus molecular weight markers were used for protein gels. Corresponding panel B displays the methylation activity of SETD2, with the loading control gel for the nuclesomes. Corresponding Panel D displays the SETD2 pulldown assay. Corresponding panel E displays the EMSA DNA gel. All relevant bands have been marked

SETD2 – K27ac EMSA

SETD2 - K36 Methylation
