# Supplementary material for: SETD2 suppresses tumorigenesis in a KRASG12C-driven lung cancer model, and its catalytic activity is regulated by histone acetylation
Source: eLife. 2025 Sep 15;14:RP107451. doi: 10.7554/eLife.107451 (PMC12435893; doi:10.7554/eLife.107451)
Supplement: Figure 5—source data 1. [file elife-107451-fig5-data1.zip › Figure 5_Source Data/Fig5E.pdf]

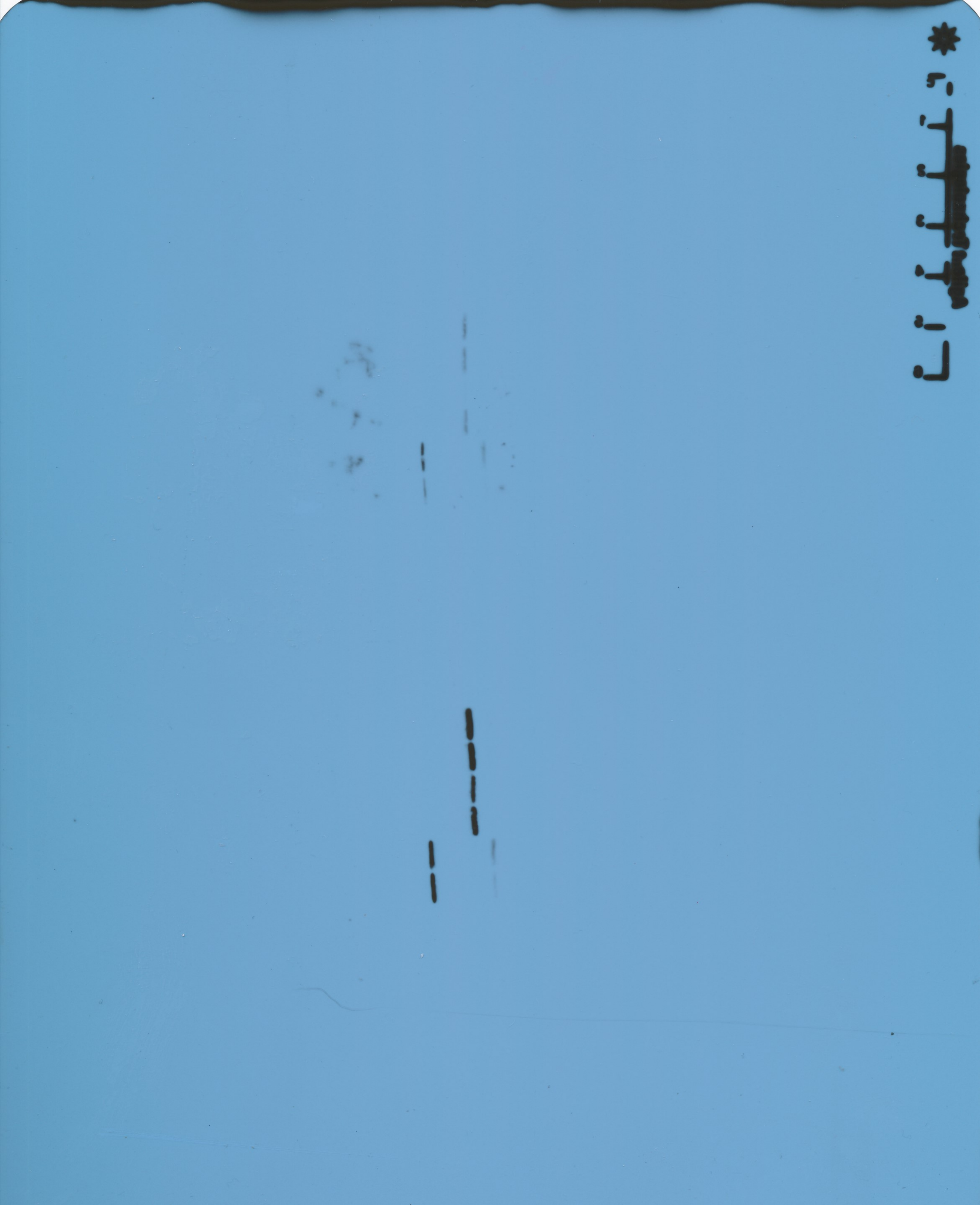

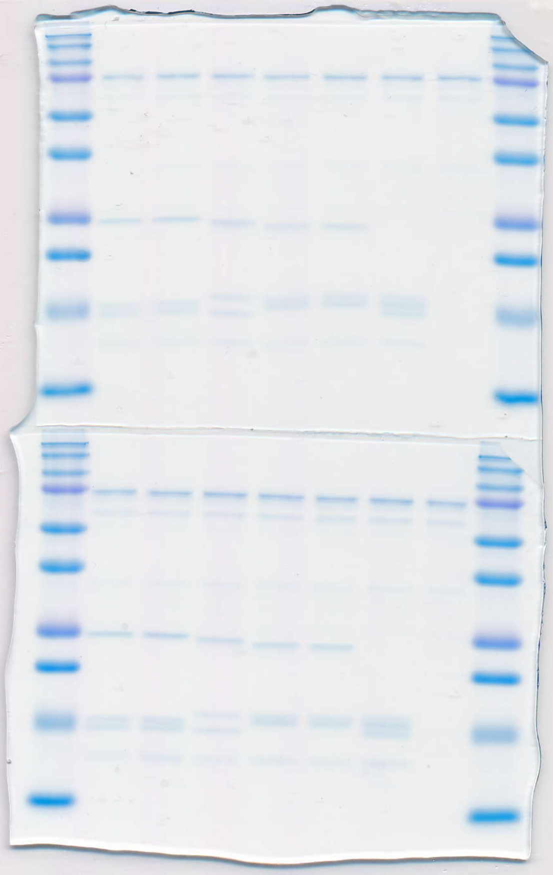

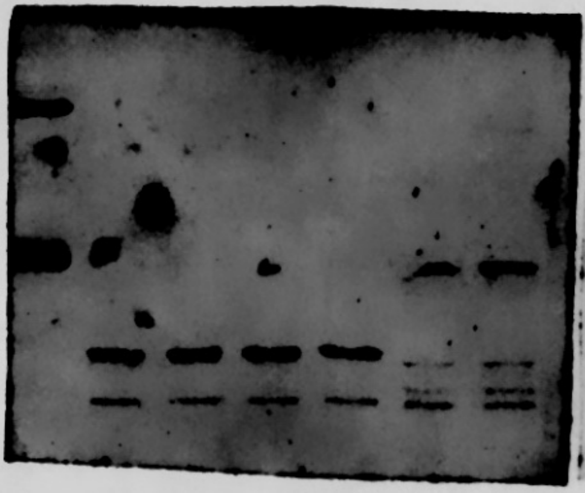

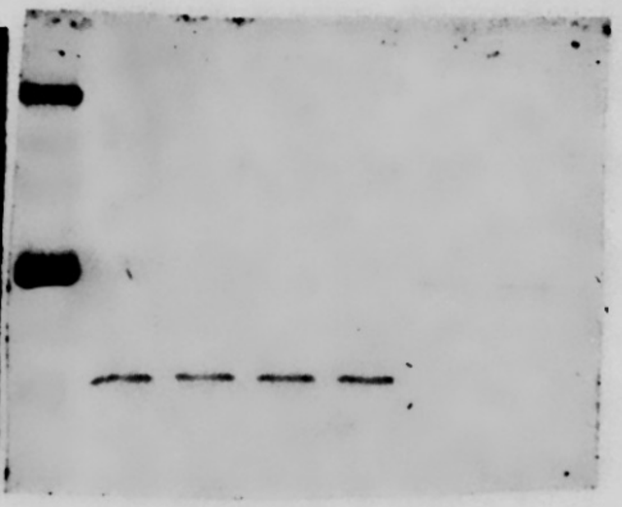

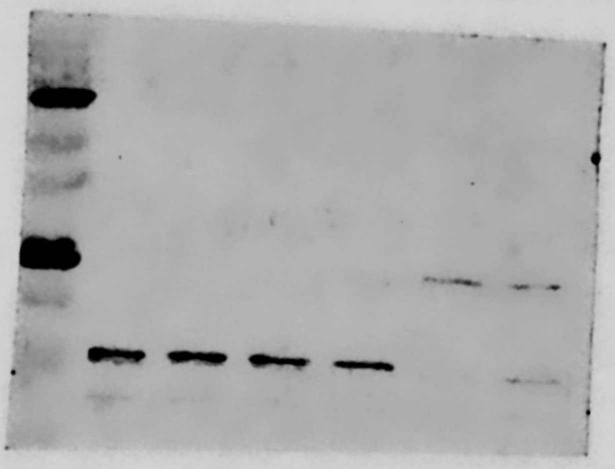

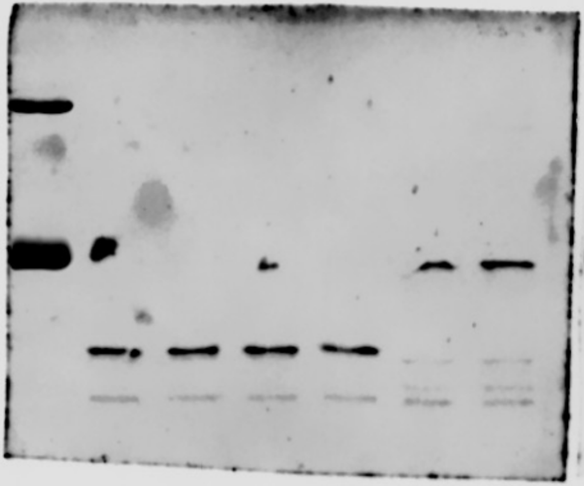
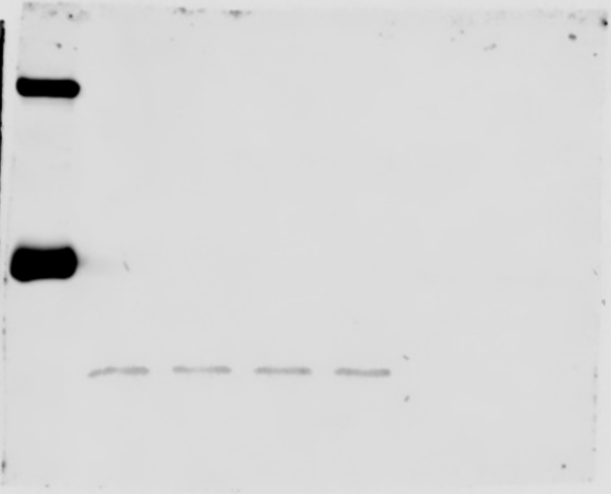
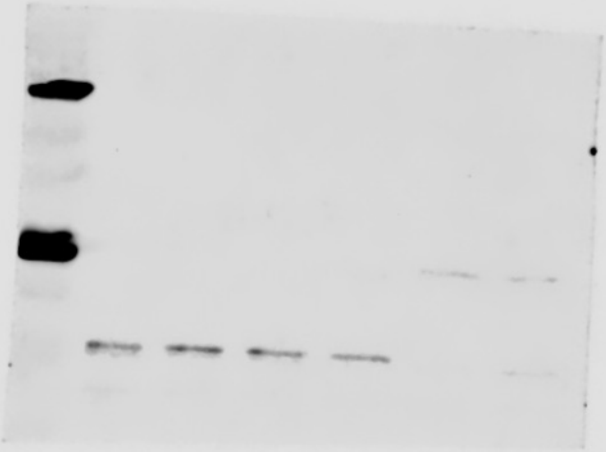

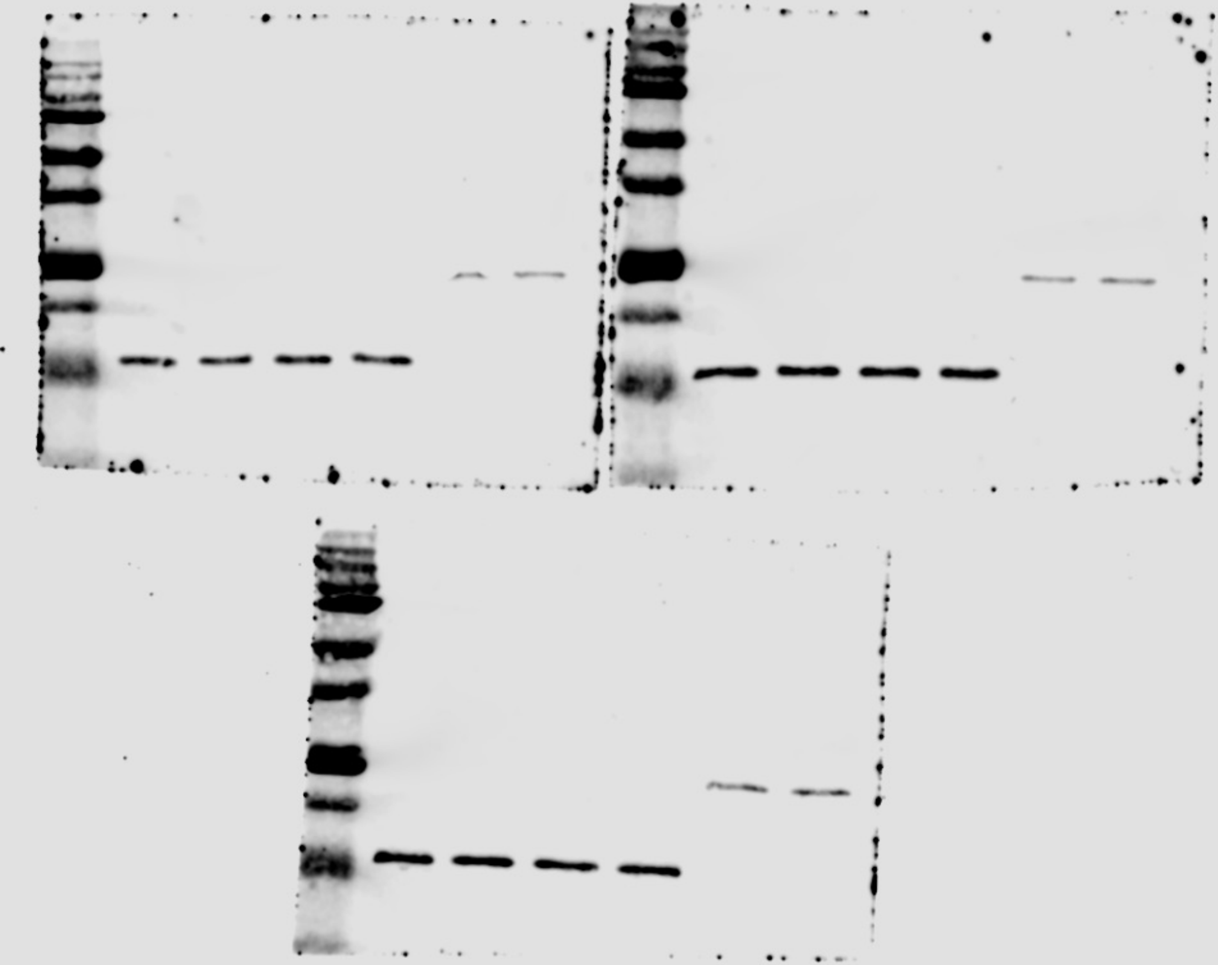

SETD2

8/11

2~day

Ub

HAc

Ø 15 119 120 14 18

Ø

Ø H2A H3 H4

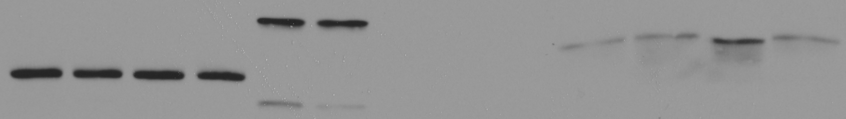

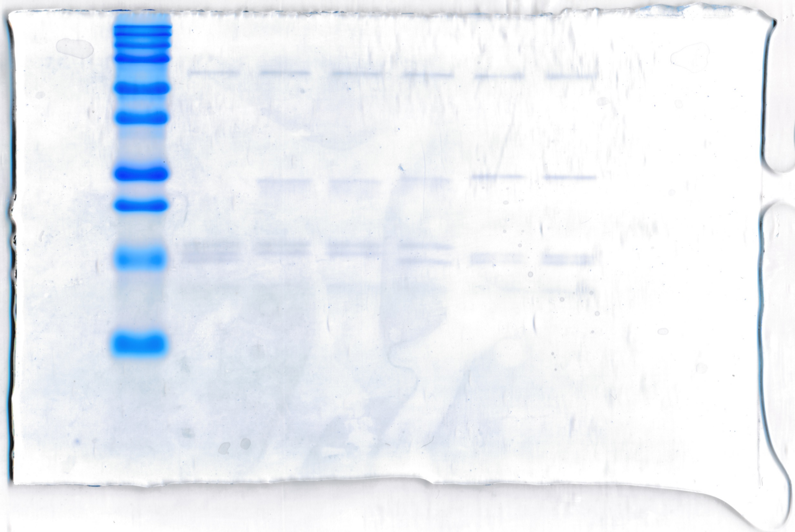

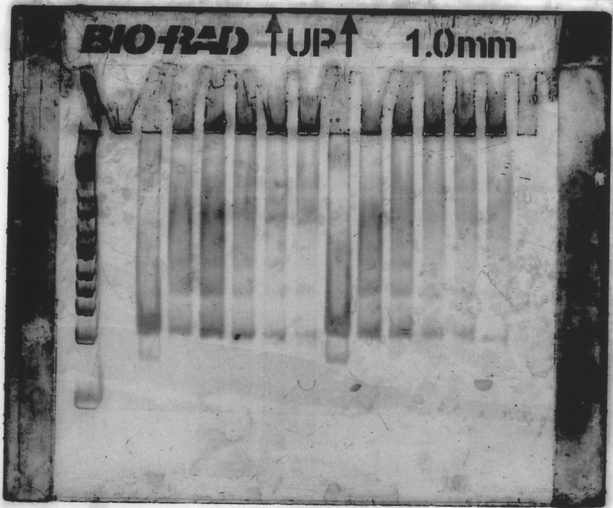

Name: Ricardo 2025-02-28 03h51m27s
Acquired: 2/28/2025 3:51:27 AM
Application: SYBR Gold

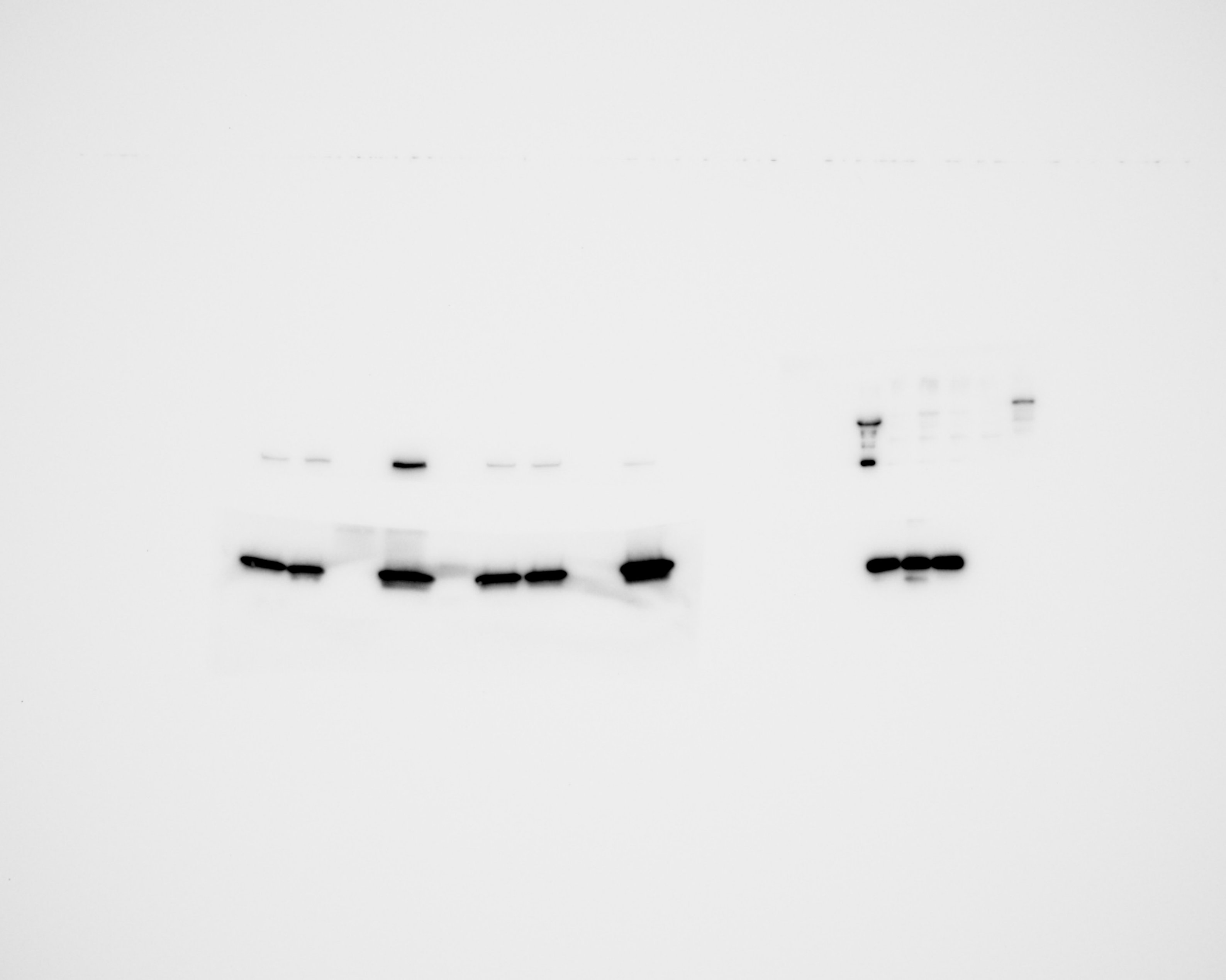

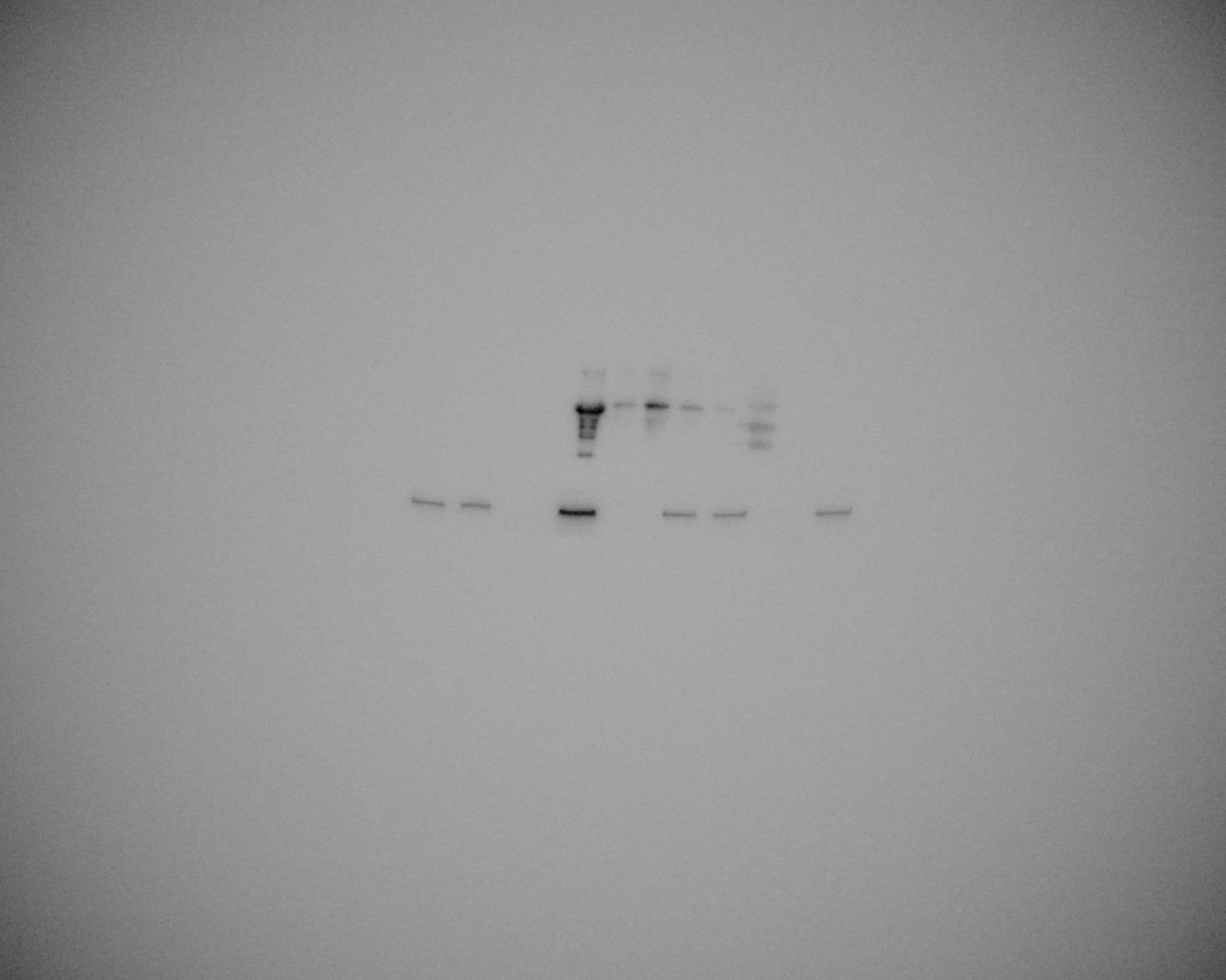

SETD2

4/9

1-day

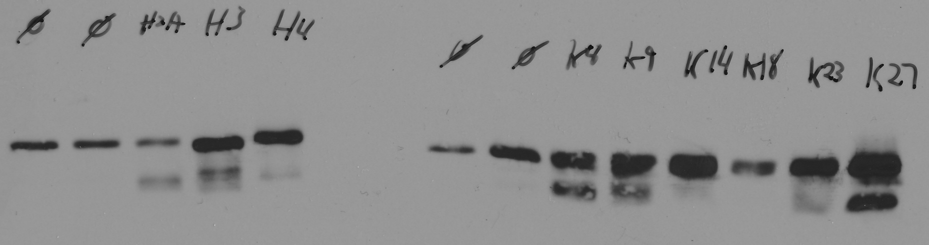

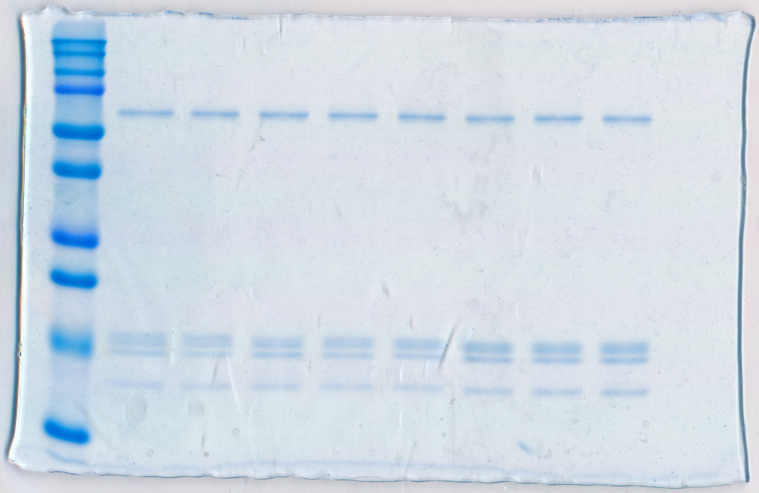

RTM

11/20

SETD2 + DOT1L

<1-day

FOP

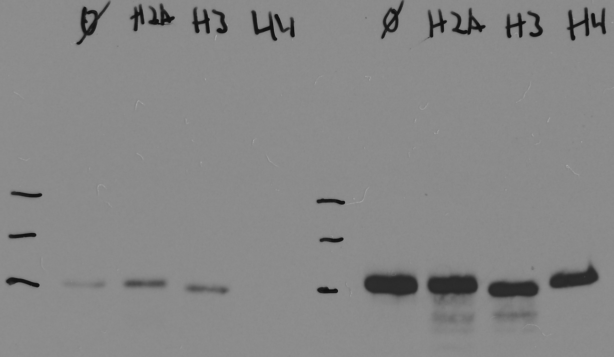

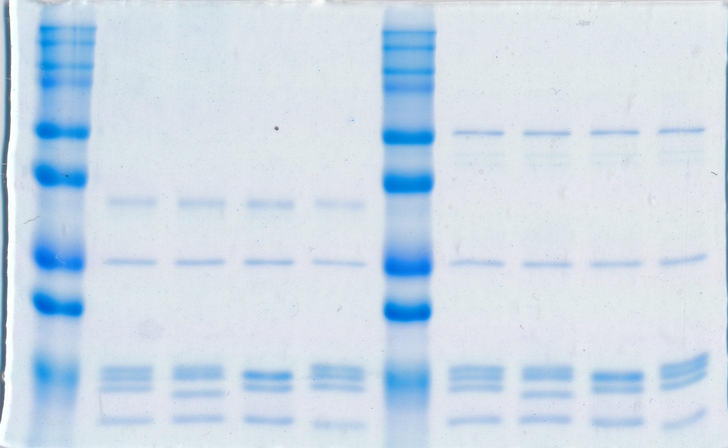

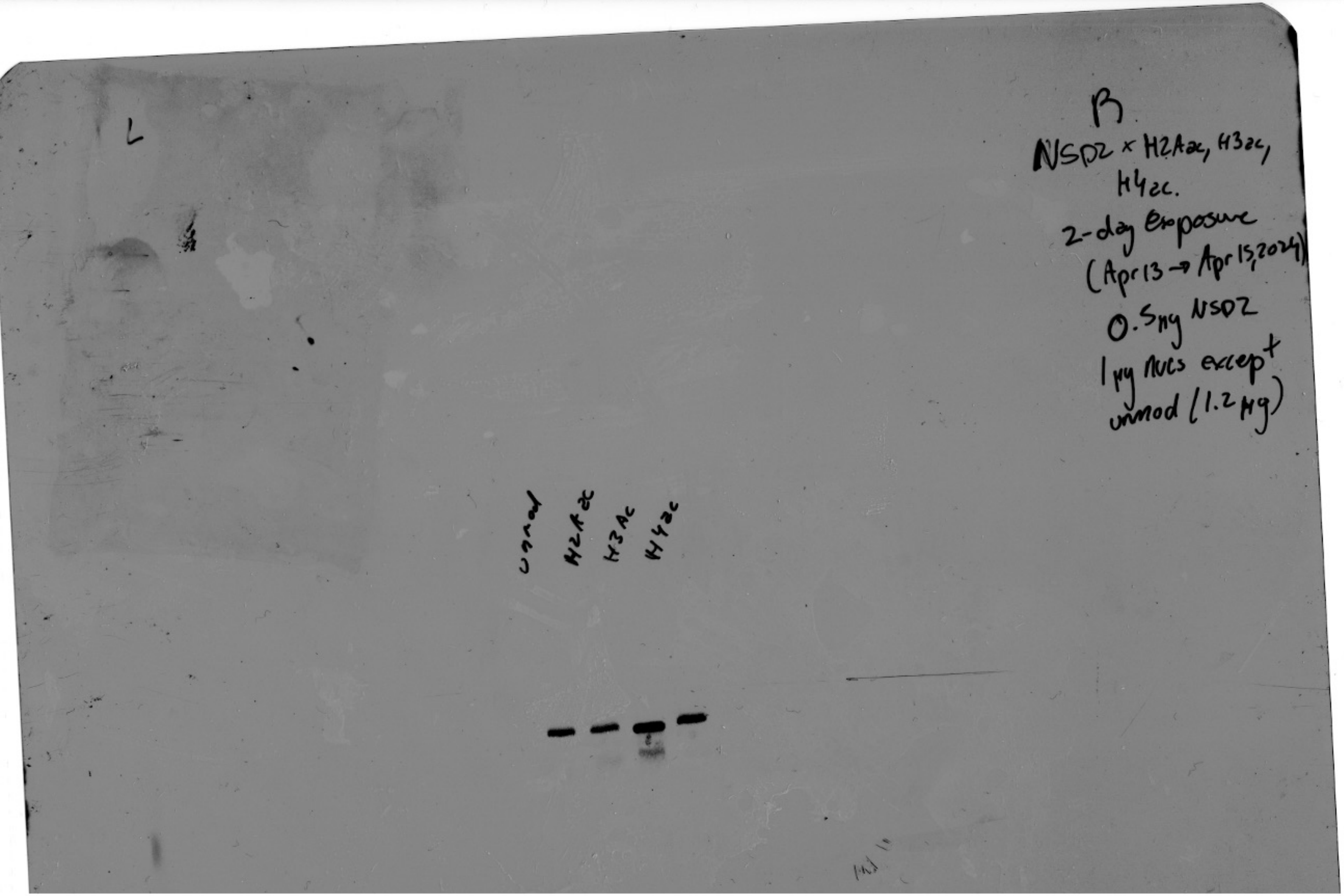
L
B
NSD2 × H2Aac, H3ac,
H4ac.
2-day exposure
(Apr 13 → Apr 15, 2024)
0.5 μg NSD2
1 μg nucs except
unmod (1.2 μg)
unmod
H2Aac
H3Ac
H4ac

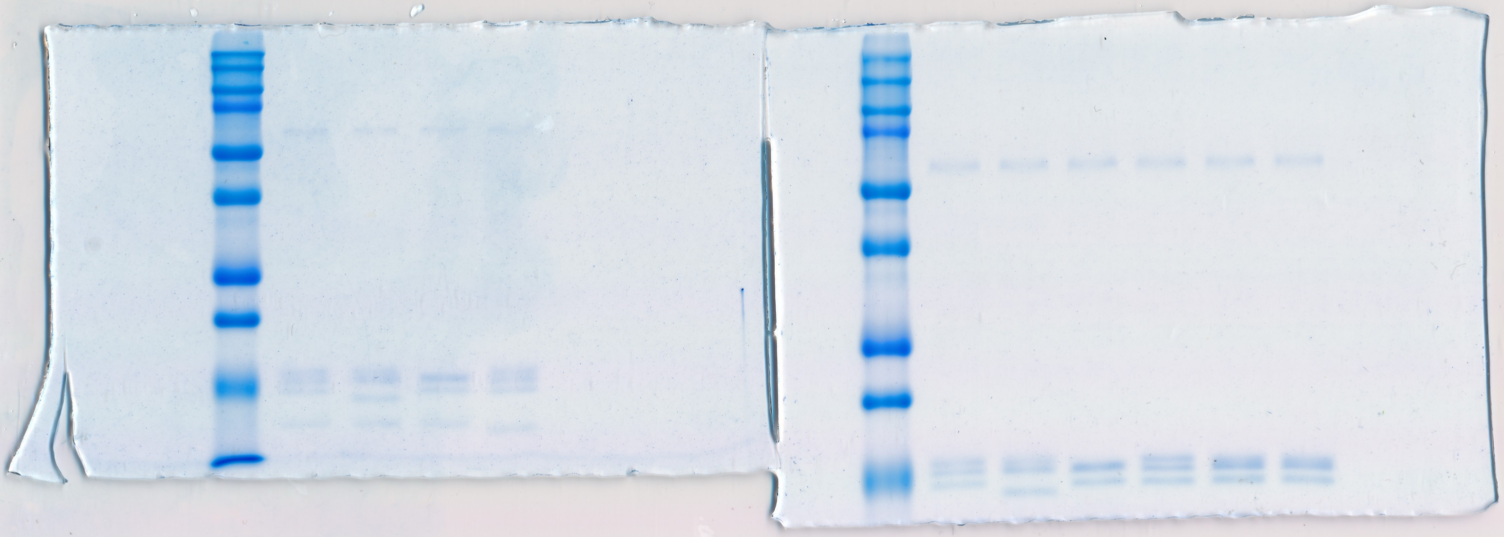

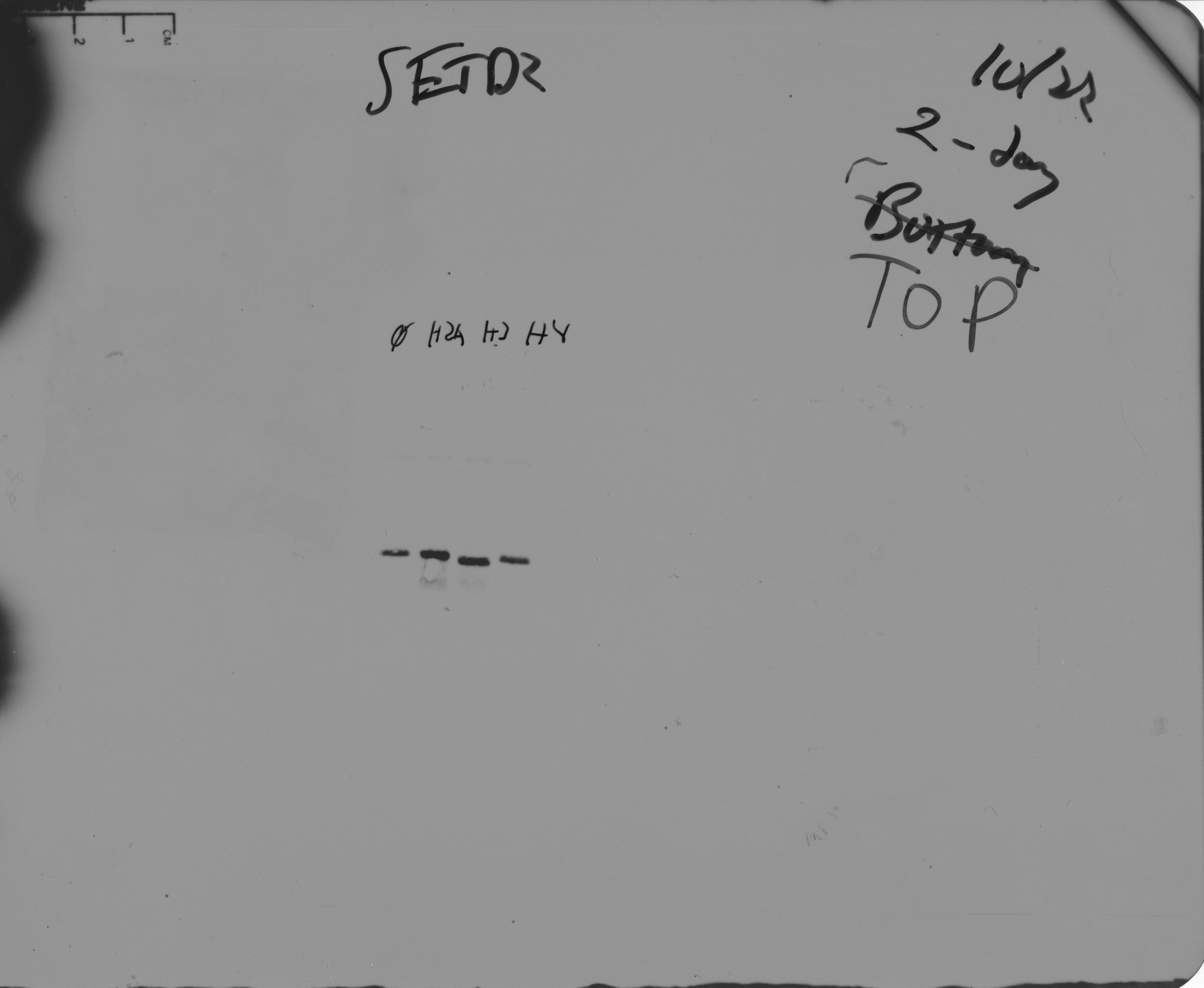
SETD2
10/22
2-day
~~Bottom~~ TOP
Ø H2A H3 H4
2 1 CM

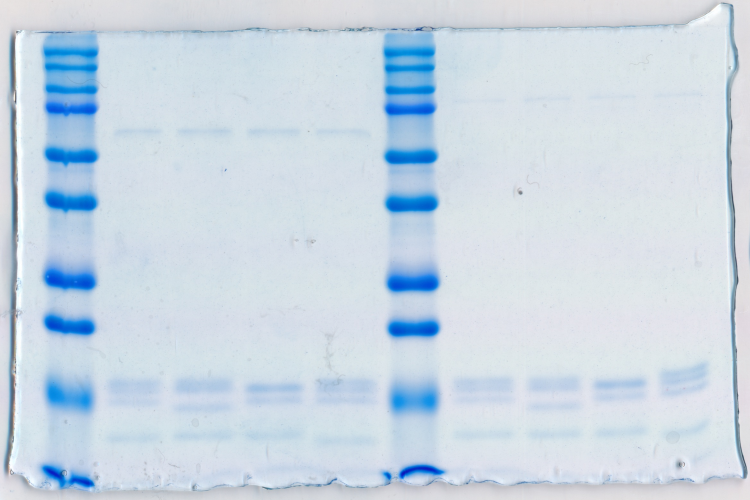

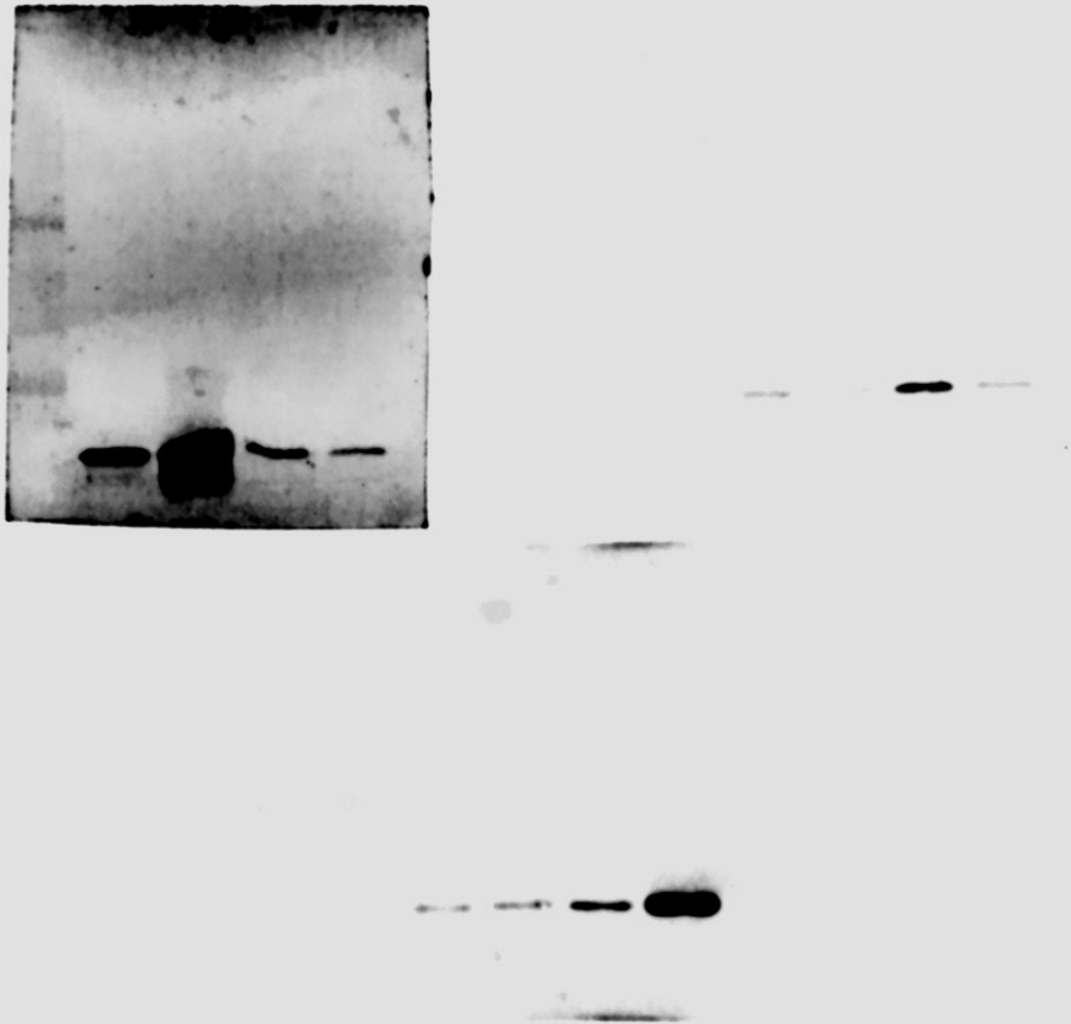

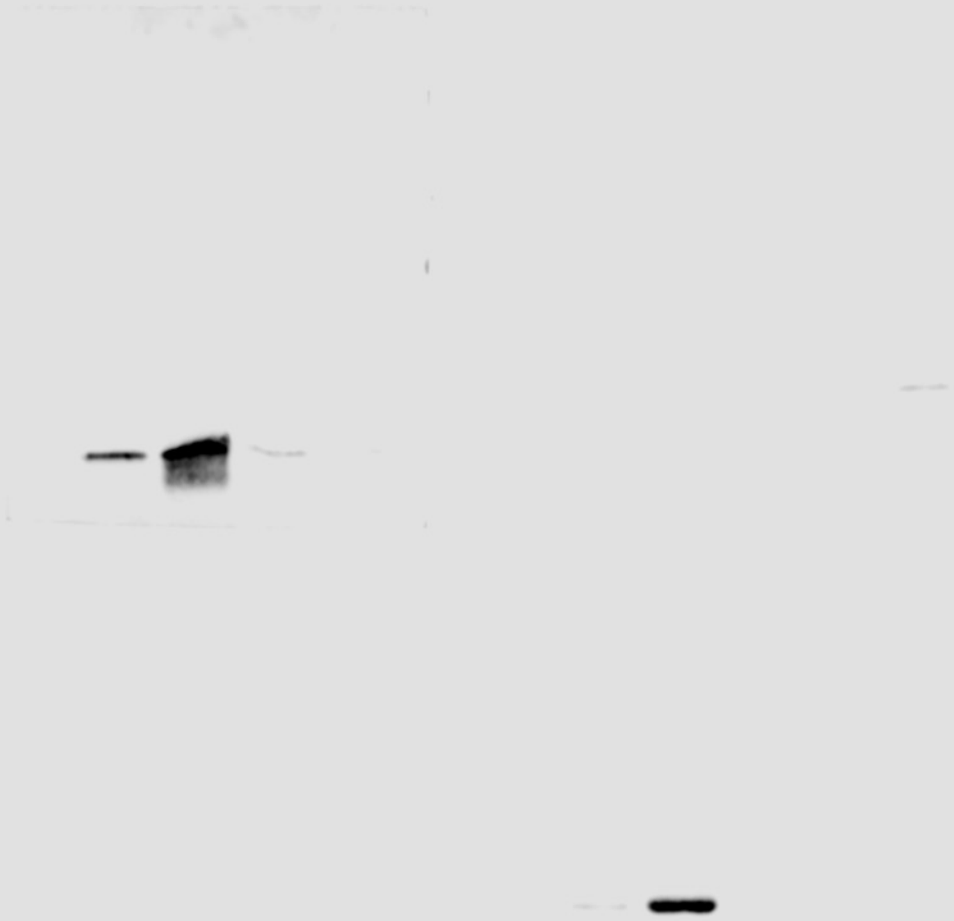

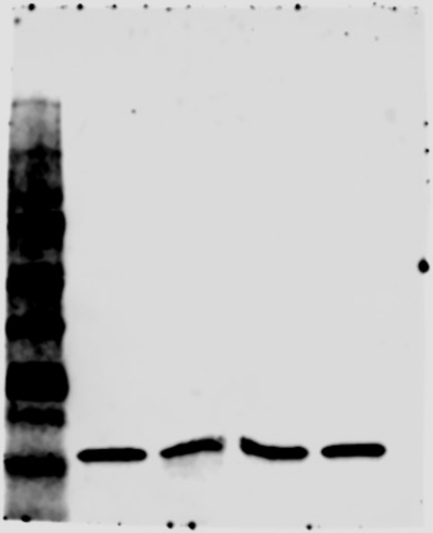

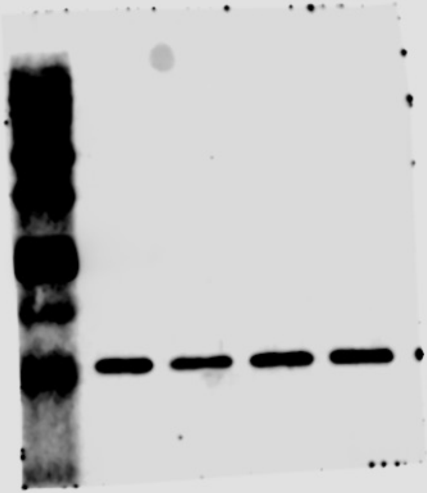

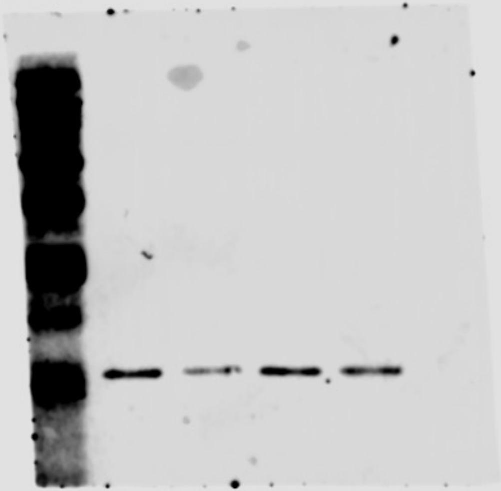

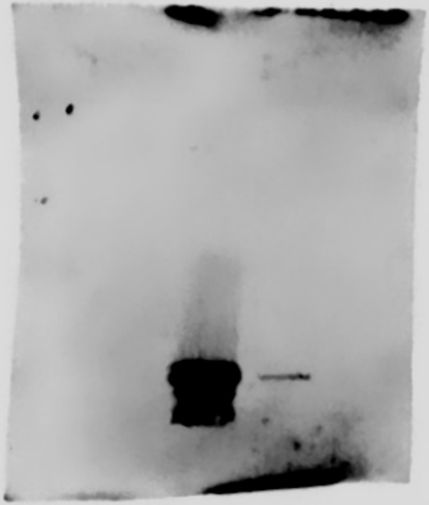
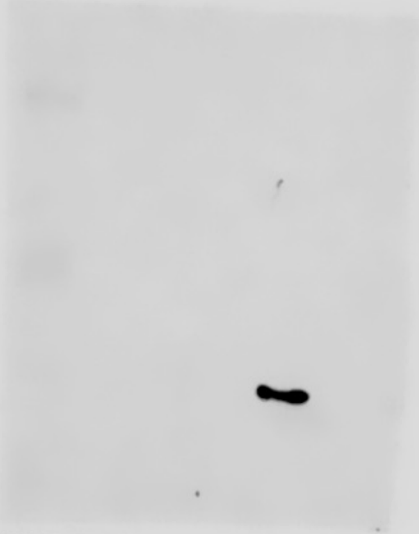
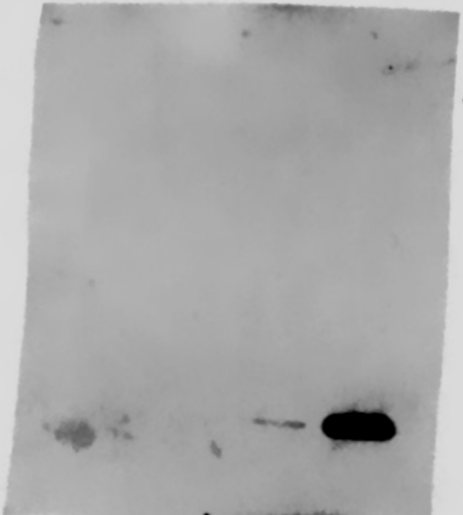

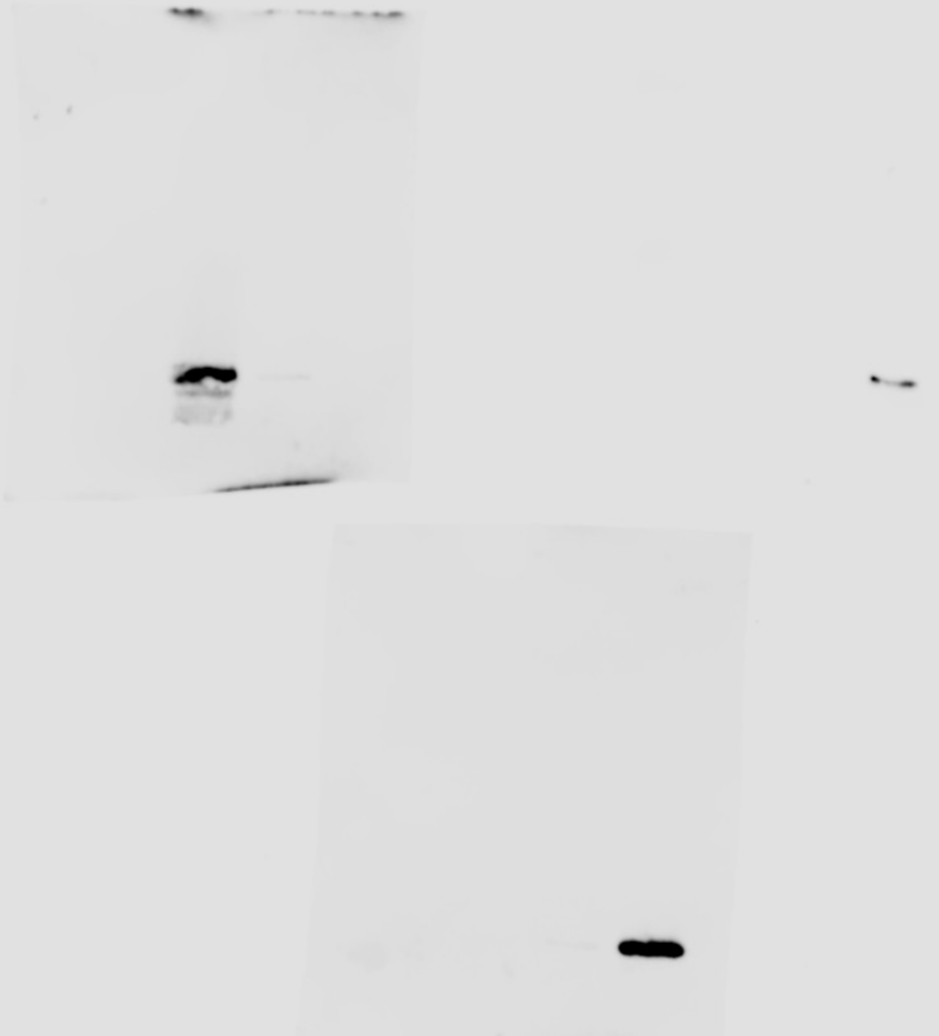

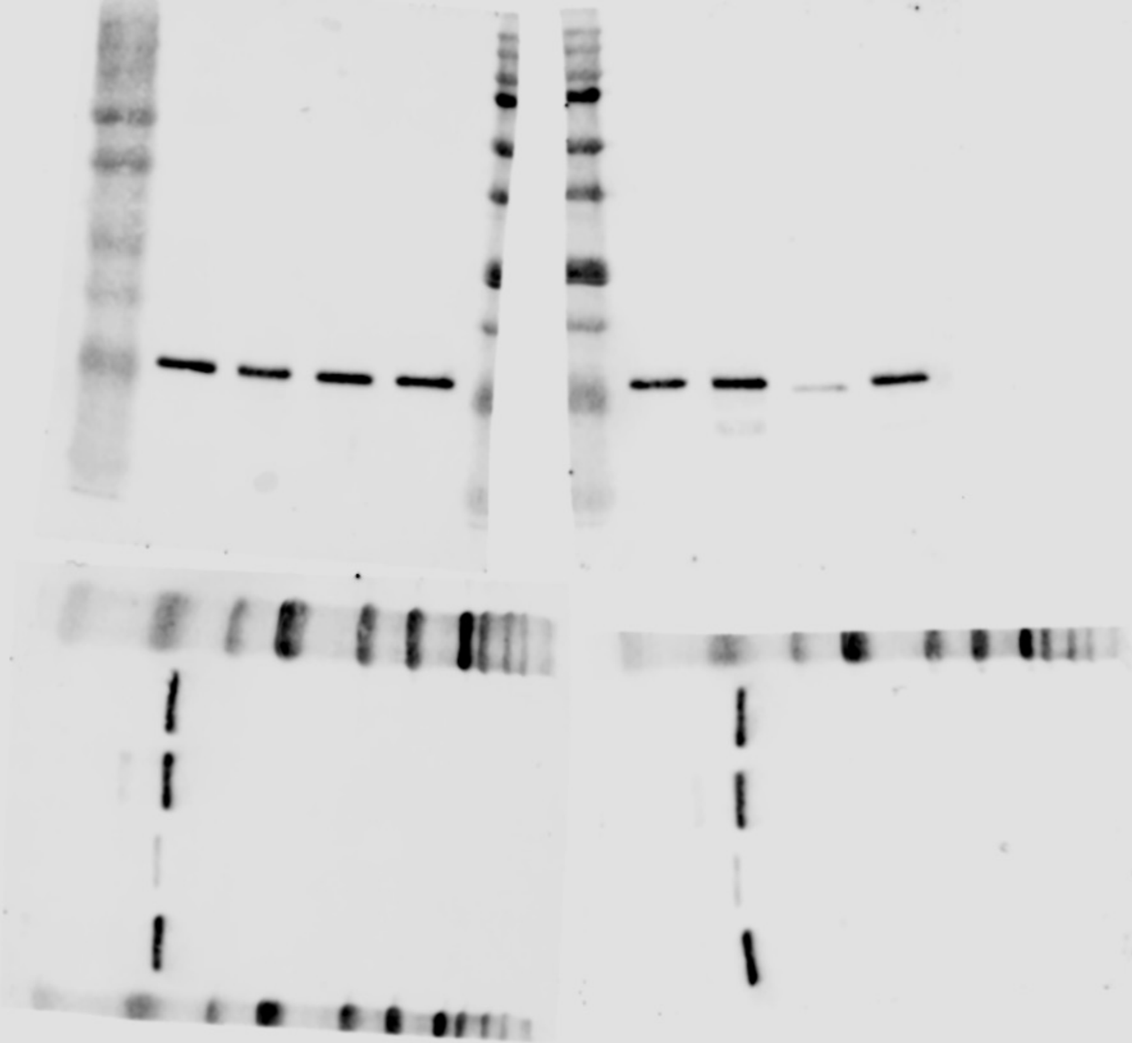

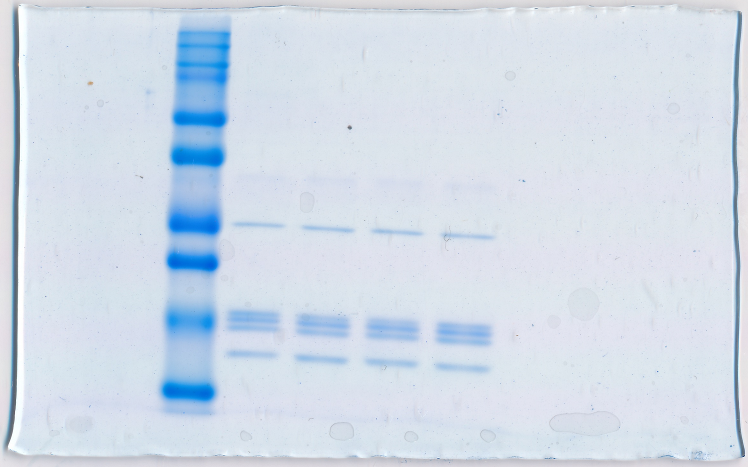

3 2 1 CM

NSD2

2/1/25

~1.5 month exposure

~~Bottom~~ middle

Ø 1 K36 2 3

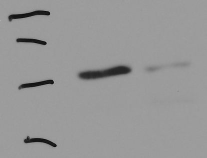

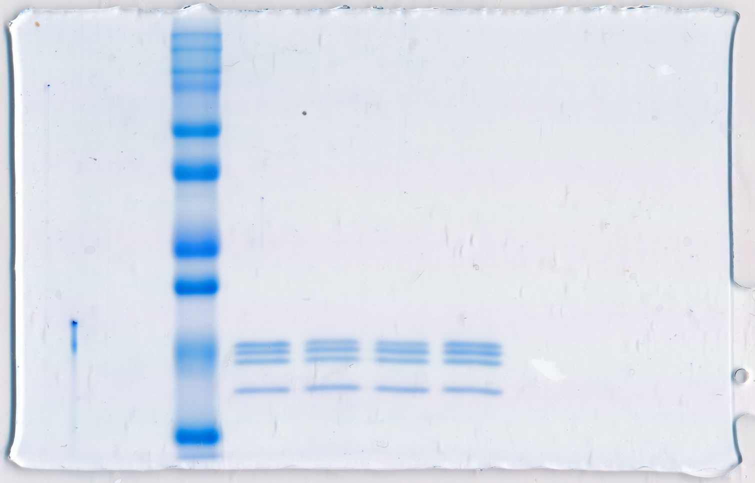

SETD2

8/2

4-day exp

U6

Ø K15 K119 120 14 18

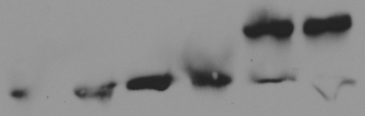

K36

Ø me1 me2 me3

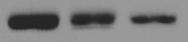

STRATAGENE

CM 1 2 3 4 5 6
