# Supplementary material for: SETD2 suppresses tumorigenesis in a KRASG12C-driven lung cancer model, and its catalytic activity is regulated by histone acetylation
Source: eLife. 2025 Sep 15;14:RP107451. doi: 10.7554/eLife.107451 (PMC12435893; doi:10.7554/eLife.107451)
Supplement: Figure 5—source data 2. [file elife-107451-fig5-data2.zip › Figure 5 SourceData_Labeled.docx]

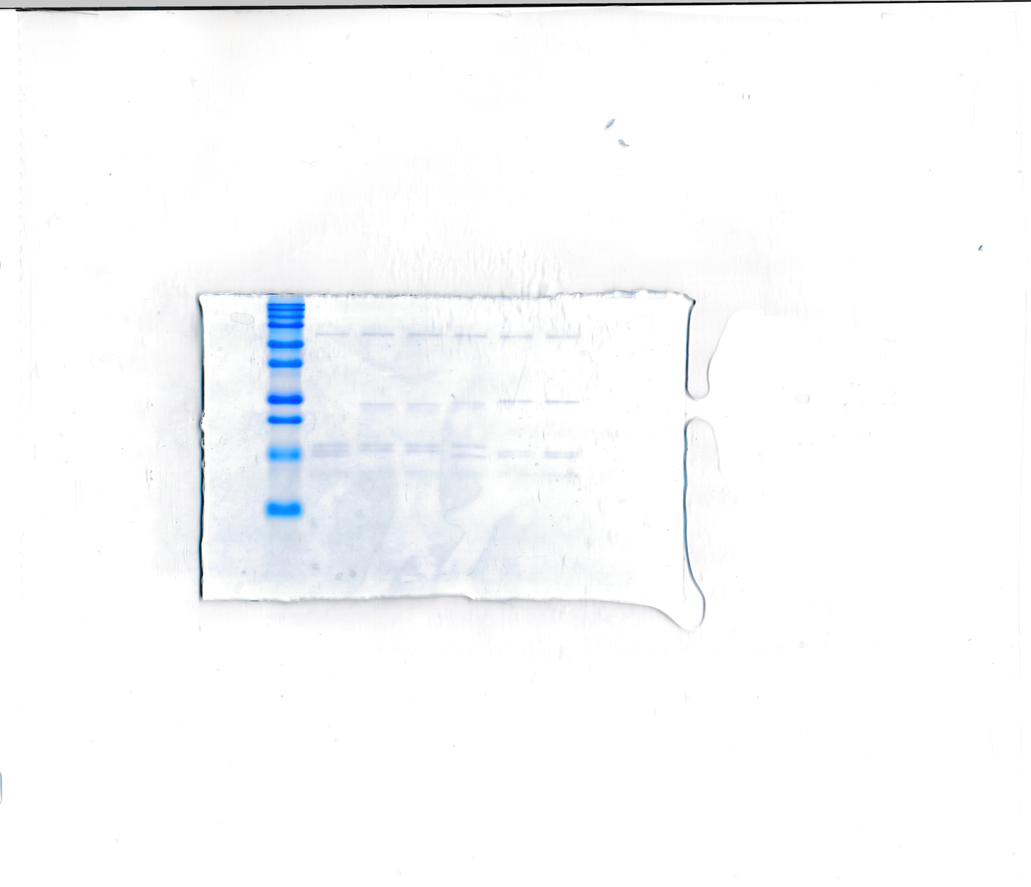

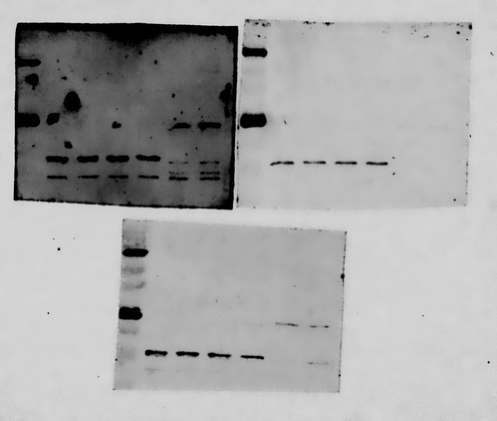

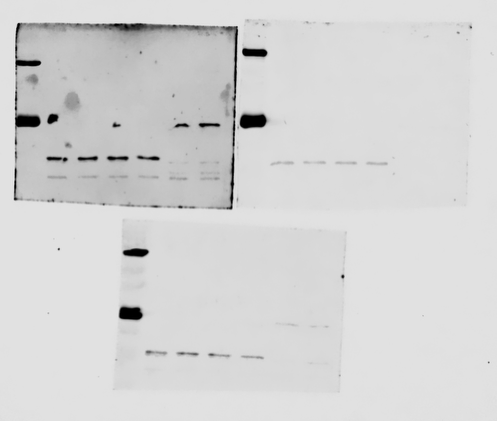

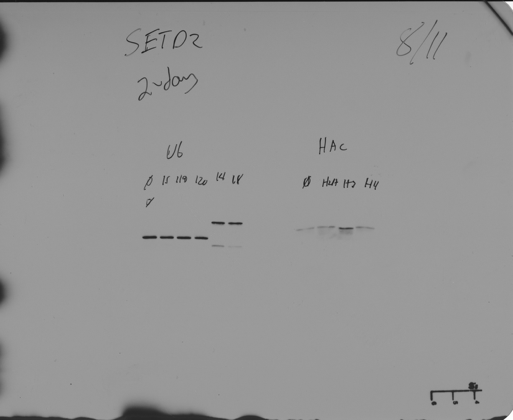

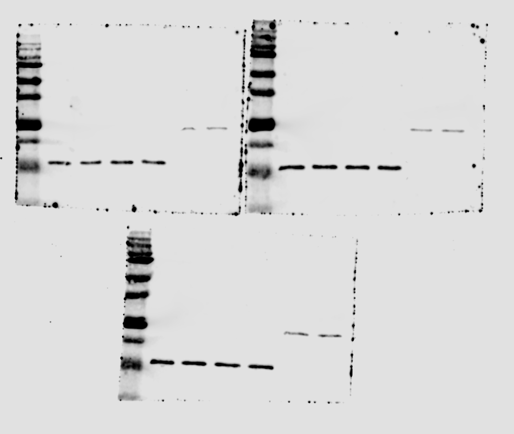


15kDa –

20kDa –

10kDa –

15kDa –

15kDa –

15kDa –

15kDa –

20kDa –

15kDa –

10kDa –

15kDa –

**Figure 5, Source Data.** Original films, gels, and membranes corresponding to Figure 5, panel B, C and E. Precision Plus molecular weight markers were used. Corresponding panels B and C display the methylation activity of SETD2 with the loading control gels for the nucleosomes. Correspnding Panel E contains methylation results for NSD2. All relevant bands have been marked.

SETD2 - K36 Methylation

NSD2 - K36 Methylation
